# Vibropolyfection: coupling polymer-mediated gene delivery to mechanical stimulation to enhance transfection of adherent cells

**DOI:** 10.1186/s12951-022-01571-x

**Published:** 2022-08-06

**Authors:** Federica Ponti, Nina Bono, Luca Russo, Paolo Bigini, Diego Mantovani, Gabriele Candiani

**Affiliations:** 1 genT_LΛB, Department of Chemistry, Materials and Chemical Engineering “G. Natta”, Politecnico di Milano, Milan, Italy; 2 Laboratory for Biomaterials and Bioengineering, CRC Tier I, Department of Min-Met-Mat Engineering and CHU de Québec Research Center, Division of Regenerative Medicine, Laval University, Quebec, QC Canada; 3 Department of Molecular Biochemistry and Pharmacology, Istituto di Ricerche Farmacologiche Mario Negri, IRCCS, Milan, Italy

**Keywords:** Gene transfer techniques, Transfection, Non-viral gene delivery, Mechanical stimulation, Polyethyleneimine

## Abstract

**Background:**

With the success of recent non-viral gene delivery-based COVID-19 vaccines, nanovectors have gained some public acceptance and come to the forefront of advanced therapies. Unfortunately, the relatively low ability of the vectors to overcome cellular barriers adversely affects their effectiveness. Scientists have thus been striving to develop ever more effective gene delivery vectors, but the results are still far from satisfactory. Therefore, developing novel strategies is probably the only way forward to bring about genuine change. Herein, we devise a brand-new gene delivery strategy to boost dramatically the transfection efficiency of two gold standard nucleic acid (NA)/polymer nanoparticles (polyplexes) in vitro.

**Results:**

We conceived a device to generate milli-to-nanoscale vibrational cues as a function of the frequency set, and deliver vertical uniaxial displacements to adherent cells in culture. A short-lived high-frequency vibrational load (t = 5 min, f = 1,000 Hz) caused abrupt and extensive plasmalemma outgrowths but was safe for cells as neither cell proliferation rate nor viability was affected. Cells took about 1 hr to revert to quasi-naïve morphology through plasma membrane remodeling. In turn, this eventually triggered the mechano-activated clathrin-mediated endocytic pathway and made cells more apt to internalize polyplexes, resulting in transfection efficiencies increased from 10-to-100-fold. Noteworthy, these results were obtained transfecting three cell lines and hard-to-transfect primary cells.

**Conclusions:**

In this work, we focus on a new technology to enhance the intracellular delivery of NAs and improve the transfection efficiency of non-viral vectors through priming adherent cells with a short vibrational stimulation. This study paves the way for capitalizing on physical cell stimulation(s) to significantly raise the effectiveness of gene delivery vectors in vitro and ex vivo.

**Graphical Abstract:**

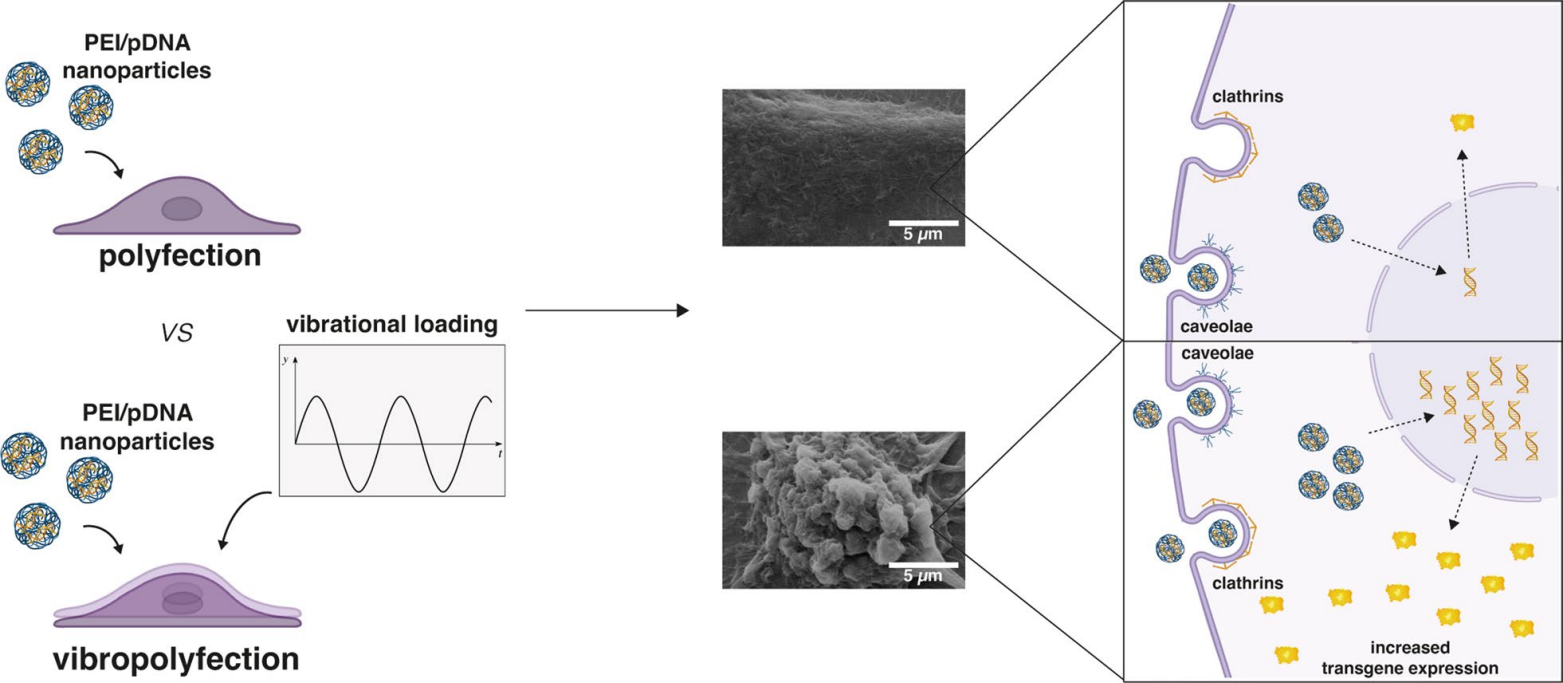

**Supplementary Information:**

The online version contains supplementary material available at 10.1186/s12951-022-01571-x.

## Background

The delivery of foreign nucleic acids (NAs) into cells and the modulation of gene expression, a process also called transfection, has emerged as a breakthrough in molecular medicine. Accordingly, NAs-based drugs have now become the leading option to study and treat many diseases through the production of recombinant proteins and set the stage for the development of advanced therapeutics and preventatives [1–3].

Transfection approaches are categorized according to the way used to transfer NAs into cells [4], named physical means and vector-mediated delivery. Broadly speaking, physical methods (e.g., electroporation, sonoporation, and optoporation) rely on the application of mechanical or electrical forces to induce the transient formation of pores in the lipid bilayer, making easier the entrance of the (naked) NA payload [5, 6]. Despite the benefits of such methods, they may lead to severe membrane damage and eventually cell death [4, 7–9].

Instead, carrier-based delivery systems (or vectors) pack and protect NAs into particles, making it easier for their delivery into cells. The clinical trial landscape has been long dominated by viral vectors, an engineered version of highly evolved viruses that naturally gain access to cells [10]. Nevertheless, some notable drawbacks have fueled the development of non-viral vectors. As a result of the increased efficacy and safety profile, they are the delivery platform utilized in Moderna’s and Pfizer/BioNTech’s COVID-19 vaccines [11–13]. Non-viral gene delivery vectors are categorized as cationic lipids (such as the key components of COVID-19 mRNA vaccines) [14] and polymers [2, 15]. These molecules naturally bind NAs and pack them into nano/micro-sized particles called lipoplexes and polyplexes, respectively, that protect the payload from the harsh extracellular environment [16]. Once gene delivery complexes are delivered to cells, they interact first with the plasma membrane and are endocytosed through distinct paths thereafter. This step is one of the most critical that stands in the way of effective NA delivery. Of note, internalization depends on both the physicochemical features of the complexes (e.g., size and surface charge) [17–19] and the cell type itself [20]. Endocytosed complexes are then entrapped within endo-lysosomal vesicles and are moved into the cytosol. To avoid exocytosis or NA unpacking and degradation, polyplexes and lipoplexes have to escape from this compartment later on [20, 21]. Unfortunately, even in the best-case scenario, only ≈ 1% of complexes meet this goal. An obvious implication is that the intracellular release of NAs is another major rate-limiting step [22]. In this context, although a tremendous effort has been made to optimize gene delivery vectors [23–28] and refine transfection technologies [22], there is still ample room for improvement.

In this work, we focus on a way to enhance the intracellular delivery of NAs and boost the transfection efficiency of widely used non-viral vectors through priming cells using mechanical stimulation. Of note, the idea of improving the effectiveness of transfectants through mechanical cell stimulation has been largely overlooked [29–34] if compared to more traditional approaches aiming to refine the vectors themselves. In the body, cells are constantly subjected to a combination of chemical and mechanical cues, which orchestrate any cell process, such as motion, proliferation, and phenotypic switching events [35, 36]. In line with this, the interaction between the cell membrane and the underlying cytoskeleton regulates the endocytic and trafficking processes [37–40]. In this light, we herein propose an in vitro cell transfection strategy relying on the combination of polymer-mediated non-viral gene delivery (hereafter referred to as polyfection) and a short-lived mechanical stimulation of cells to temporarily control their behavior. Drawing from some seminal works on cell response to high-frequency nanoscale cues [41, 42], we developed a stimulation device that applies micro-to-nanovibrations perpendicular to the plane of the cell culture plate to induce reversible plasma membrane remodeling. The *gold standard* transfectant polyethyleneimine (PEI) [2, 43, 44], in the linear (*l*PEI) and branched (*b*PEI) structure, was used to convey NAs into mechanically-stimulated cells. Priming cells with short-lived high-frequency mechanical load allowed triggering clathrin-mediated endocytosis and improved the uptake of gene delivery particles. This underpinned a significant enhancement of transfection efficiency.

## Results and discussion

### Assessment of the functioning of the vibrational-loading device

**Fig. 1:**
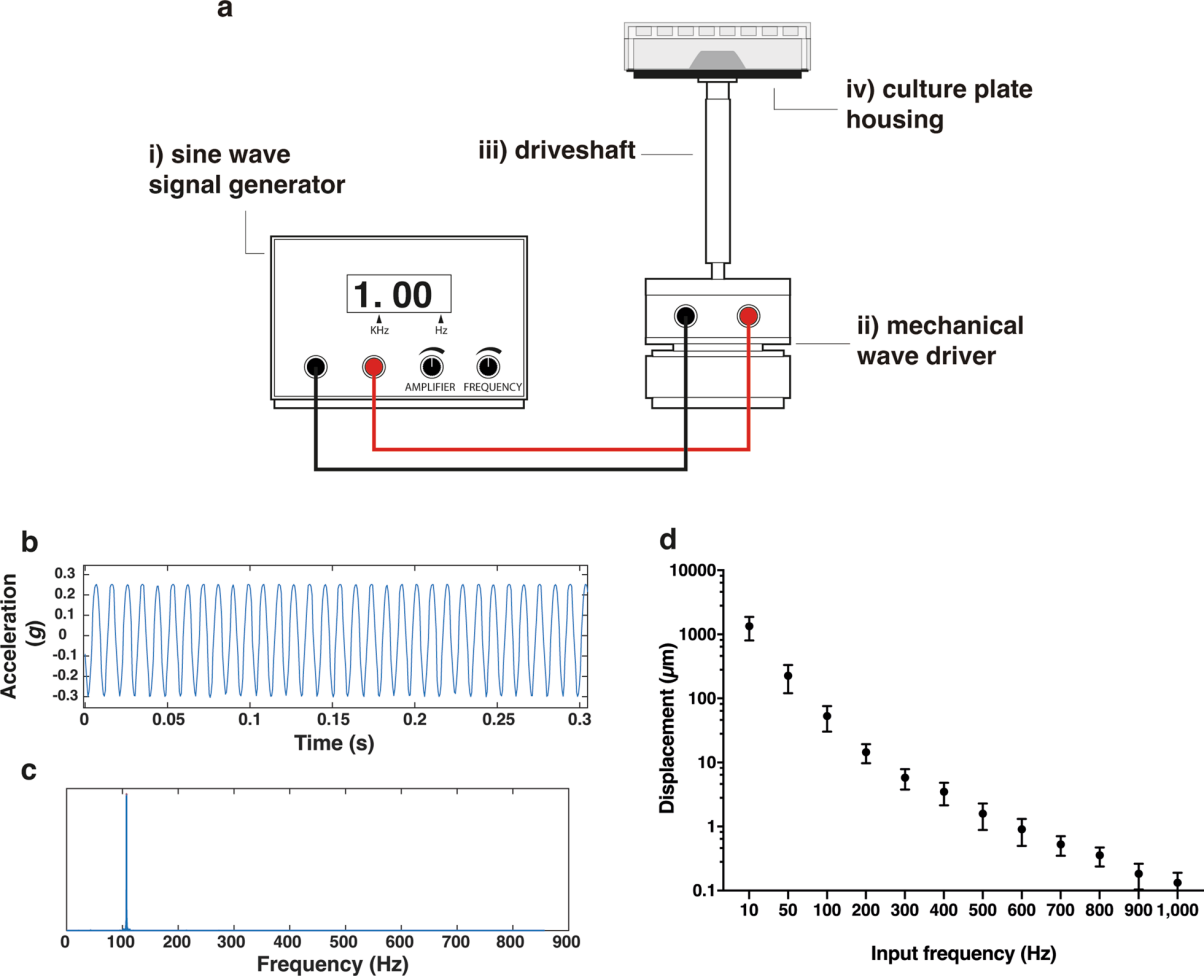
**a** Schematic representation of the vibrational-loading device. The device consists of i) a sine wave signal generator able to modulate the input frequency (f_in_) from 0.1 Hz to 10,000 Hz, ii) the mechanical wave driver, and iii) the driveshaft able to move along the z-axis. This is equipped with iv) a built-in-home cell culture plate housing on the top. **b** Plot of z-axis acceleration signals acquired at f_in_ = 100 Hz (raw data in the time domain) and **c** the outcomes obtained after Fast Fourier Transformation (FFT) in the frequency domain. **d** Displacement values of the motion shaft on the z-axis as a function of the input frequency applied (f_in_). Data are expressed as mean ± SD (n = 3)

A vibrational-loading device was conceived to apply sinusoidal micro-to-nanoscale displacements to adherent cells in culture. The device is enabled to exert vertical displacements (along the z-axis) of a motion shaft utilizing a sine wave generator (please refer to the Methods section for the detailed description of the stimulation device). Adherent cells were seeded in a culture plate that was integral to the driveshaft (Fig. 1a). Because of this, the cells experienced the vibrational loadings applied. The displacements caused by the input frequencies (f_in_) set on the sine wave generator were characterized using an accelerometer. Figure 1b shows the acceleration data obtained at f_in_ = 100 Hz from the recordings on the time domain and the corresponding outcomes obtained after Fast Fourier Transformation (FFT) in the frequency domain (Fig. 1c). As expected, the higher the sinusoidal wave frequencies applied, the smaller the displacement of the culture plate. As depicted in Fig. 1d, the displacements decreased from the millimeter (1.3 ± 0.7 mm) to the nanometer (99.0 ± 0.1 nm) range as the f_in_ applied was increased from 10 Hz to 1,000 Hz. Of note, there was no significant acceleration on the x- and y-axis (data not shown), meaning that the motion of the drive arm was actually controllable and uniaxial.

In short, this device allowed controlling the generation of milli-to-nanoscale vibrational cues depending on the different f_in_ set. This is a key issue since it is widely known that displacements arising from high-frequency vibrations can be profitably used to control cell behavior [41, 45].

### Assessing cell response to vibrational loading

The basic requirement that any cell-stimulation technology has to comply with is that mechanical cues shall not cause lethal or even cytotoxic effects [35, 36]. We addressed this issue on three well-established adherent cell lines (L929, HeLa, MG-63 cells), which are also very permissive to transfection [2, 46, 47], and hard-to-transfect bovine articular primary chondrocytes (bAPCs) [48].

**Fig. 2:**
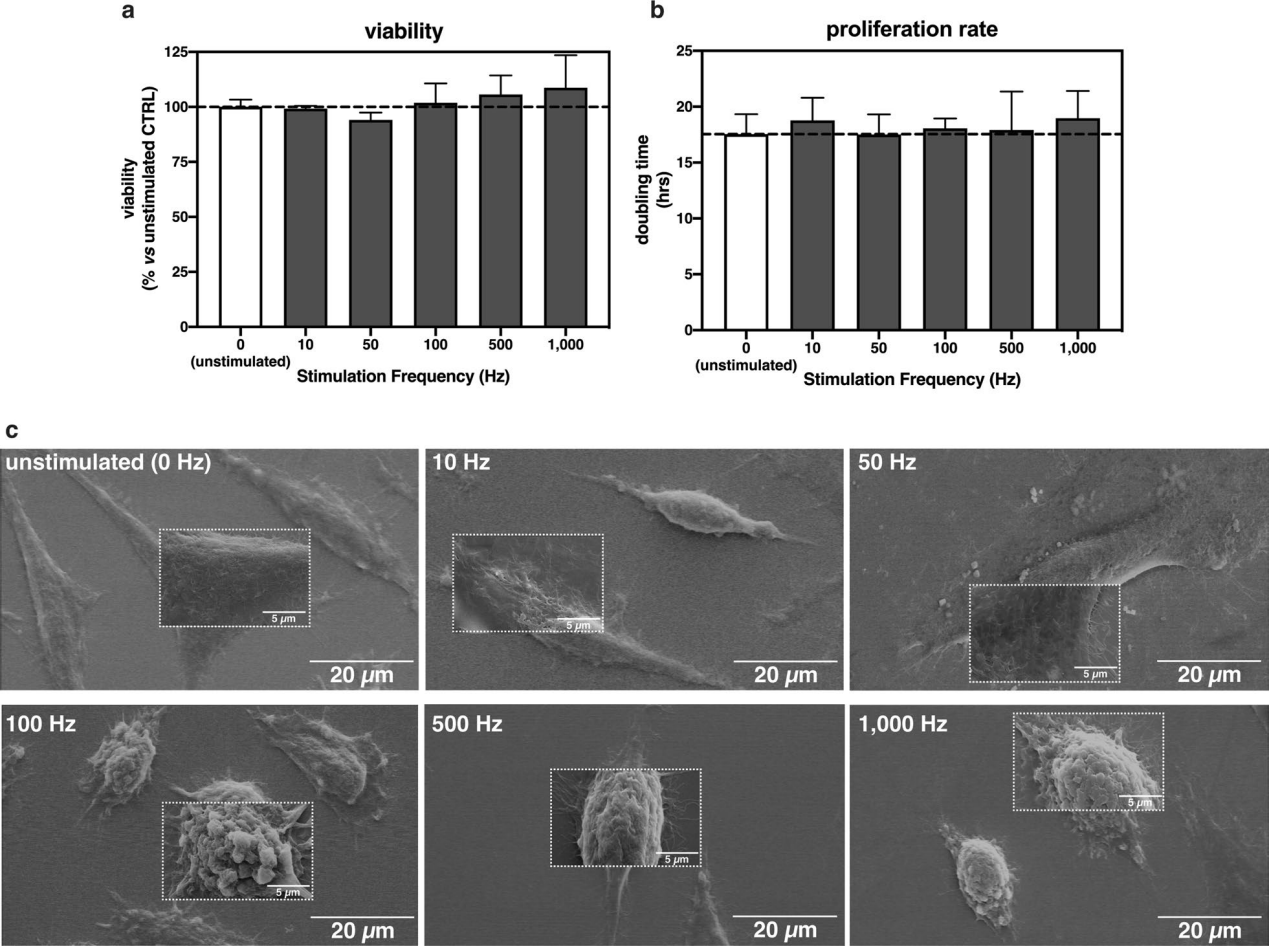
Effect of a 5 min-long vibrational loading on the viability, proliferation rate, and morphology of L929 cells. **a** Viability and **b** doubling time of L929 cells that underwent vibrational loading for 5 min at f_in_ = 10 Hz, 50 Hz, 100 Hz, 500 Hz, and 1,000 Hz. Results are expressed as mean ± SD (n = 3). Unstimulated cells were kept as controls, and their viability (100%) and doubling time (18 hrs) as baselines. **c** Representative SEM micrograph of L929 cells undergoing vibrational loading for 5 min at f_in_ = 10 Hz, 50 Hz, 100 Hz, 500 Hz, and 1,000 Hz. Unstimulated cells were kept as controls. Larger background images were taken at × 1,500 (scale bar = 20 µm) and inset micrographs at ×7,000 magnification (scale bar = 5 µm)

First, the viability of adherent L929 cells was evaluated upon a 5 min-long vibrational loading. Interestingly, there were no changes in the cell viability as a function of the wave frequency applied (Fig. 2a) (p > 0.05 vs. unstimulated cells). It is worthy of note that the same applies to the other cell lines, namely HeLa, MG-63, and bAPCs (Additional file 1: Fig. S1a).

Furthermore, as plasmid DNA (pDNA) could gain access to the nucleus during mitosis and be transcribed thereafter [49], landmark studies have pointed to the cell proliferation rate as a key parameter ruling the transfection effectiveness [2, 49, 50]. Indeed, there is evidence that mechanical triggers may induce disparate changes in cell behavior [41, 51]. Therefore, we checked for possible modifications in cell proliferation rates following mechanical stimulation. As follows from Fig. 2b, a 5 min-long vibrational loading at any given stimulation f_in_ tested induced no significant changes in the cell doubling time.

Vibrational stimuli have been traditionally used to direct the differentiation of mesenchymal stem cells into osteoblasts, and control the adhesion, migration, and proliferation of endothelial cells [41] as a result of cytoskeletal remodeling and the mechanotransduction-driven regulation of target genes [52, 53]. To shed light on the possible effect(s) of the vibrational loading on the sole gene delivery process, we used Scanning Electron Microscopy (SEM) to take a close-up view of the cell surface upon the application of micro- and nano-vibrational stimuli. Because plasmalemma defines cell boundaries, it is the primary barrier that stands in the way of introducing any exogenous payload into the cell. In this case, a short-lived vibrational loading of 5 min at specific wave frequencies (f_in_ ≥ 100 Hz) did elicit gross cell morphological changes (Fig. 2c). As opposed to unstimulated controls that appeared markedly smoother, stimulated cells at f_in_ ≥ 100 Hz exhibited a rough surface, densely studded with membrane protrusions, which are blister-like outgrowths of the plasma membrane that may occur upon membrane detachment from the underlying cytoskeleton in diverse circumstances [54]. This was the first tangible evidence that one can avoid triggering relevant cell modifications through high-frequency loads. Again, the mechano-induced morphological changes were consistent across the different cell types examined (Additional file 1: Figs. S1b–d). Of note, plasma membrane outgrowths were in the range of 0.7 µm ÷ 1 µm in diameter (Additional file 1: Table S1). These closely match the dimensions of blebs, spherical membrane evaginations that form in response to different stimuli [55, 56].

Besides, no perforations were visible on the plasma membrane of stimulated cells, unlike what typically occurs to cells subjected to electroporation or sonoporation [57]. Altogether, these results pointed to the potential of using a short-lived vibrational loading to induce abrupt membrane rearrangements in a variety of mammalian cells and disclosed a f_in_ of 100 Hz as the threshold to trigger such modifications.

**Fig. 3:**
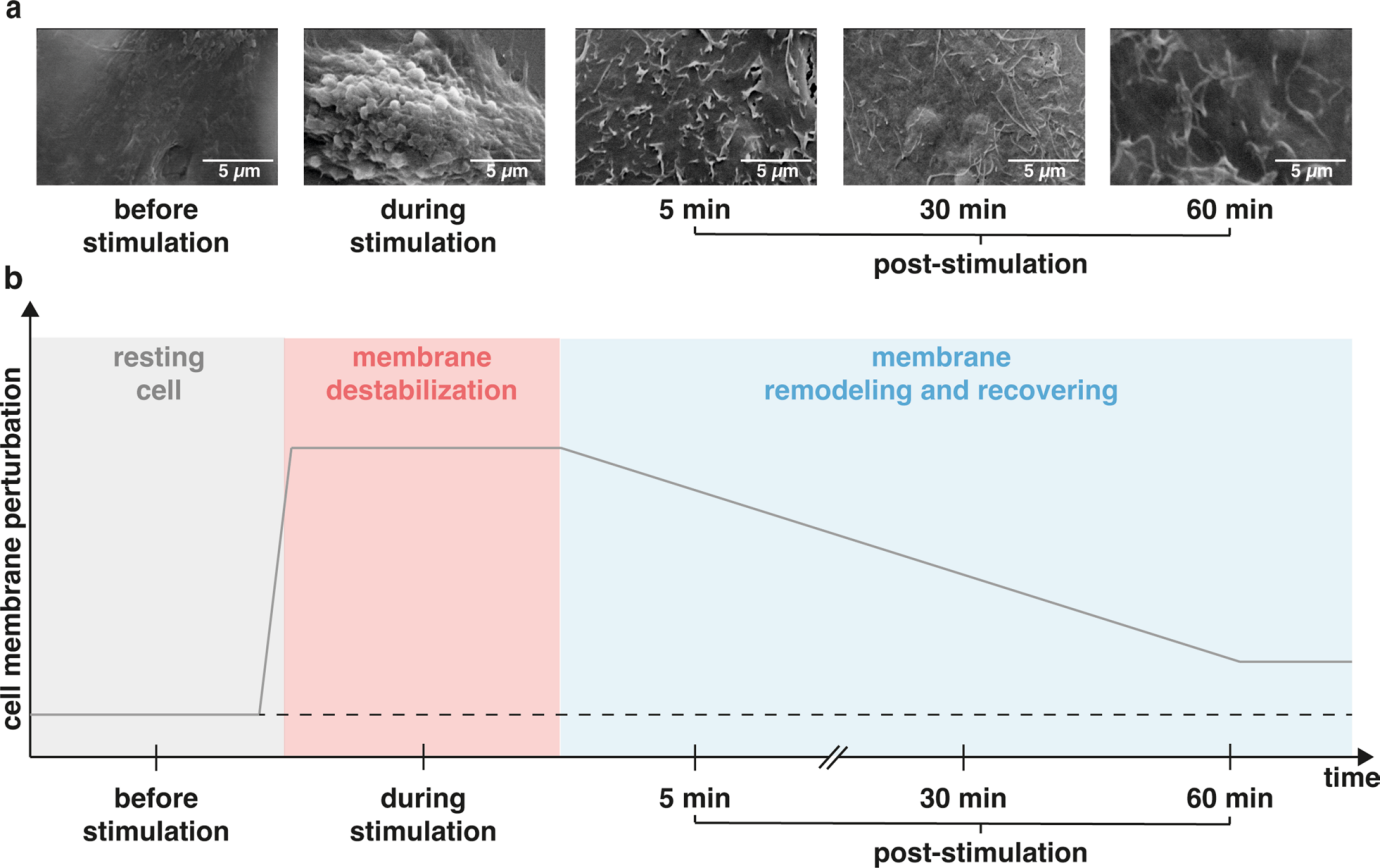
Membrane recovery kinetics in vibration-loaded cells. **a** SEM micrographs of L929 cells were taken before stimulation, during stimulation at f_in_ = 500 Hz, and at 5 min, 30 min, and 60 min after discontinuation of the vibrational loading. Images were taken at ×7,000 magnification (scale bar = 5 µm). **b** Temporal evolution of cell membrane disruption and recovery upon vibrational loading. The extent of cell membrane perturbation is purely by way of example

On the other hand, mechanical triggers can destabilize the cell membrane, thus leading to unintended cell death. This shortcoming applies to several drug delivery methods, such as sonoporation [4, 58], electroporation [ 59–61], optoporation [62], and also ours. At the end of stimulation, cells are thus being called upon to act to survive, meaning that they have to remodel their disrupted membrane as early as possible. Therefore, we decided to monitor the membrane recovery kinetics upon vibrational stimulation of cells. In response to abrupt plasmalemma ruffling/blebbing (Figs. 2c, 3a), cells mostly reverted to normal morphology within about 60 min (Fig. 3).

When cells are called to action in response to membrane perturbations, local and passive membrane rearrangements take place first (Fig. 3). Next, a second, long-lasting remodeling takes over. During this time, the cells attempt to restore their naïve morphology through the de novo synthesis of cytoskeletal elements, and extensive membrane trafficking processes [63]. The rightmost of Fig. 3 shows that 1 hr post-stimulation cells have not fully recovered the original morphology because the healing process takes longer [64, 65]. Although blebs were traditionally viewed as a sign of apoptosis, there is an increasing body of evidence supporting their active role in cell motility and spreading [66], mitosis [55], and endocytosis [67]. During the formation of blebs, as the membrane tension increases, the overall endocytic vesicle formation is slowed down. This implies that the endocytic mechanisms may occur as part of the bleb retraction process [68], when the decrease of membrane tension leads to increased endocytic rates [69]. On the other hand, it is worth mentioning that membrane tension is just one of the factors affecting mechanosensitive endocytosis [70].

In light of the above, we hypothesized that mechano-induced membrane destabilization could be harnessed to enhance membrane trafficking and improve the delivery of NAs into cells.

### Effectiveness and stability of polyplexes on cells undergoing vibropolyfection

**Fig. 4:**
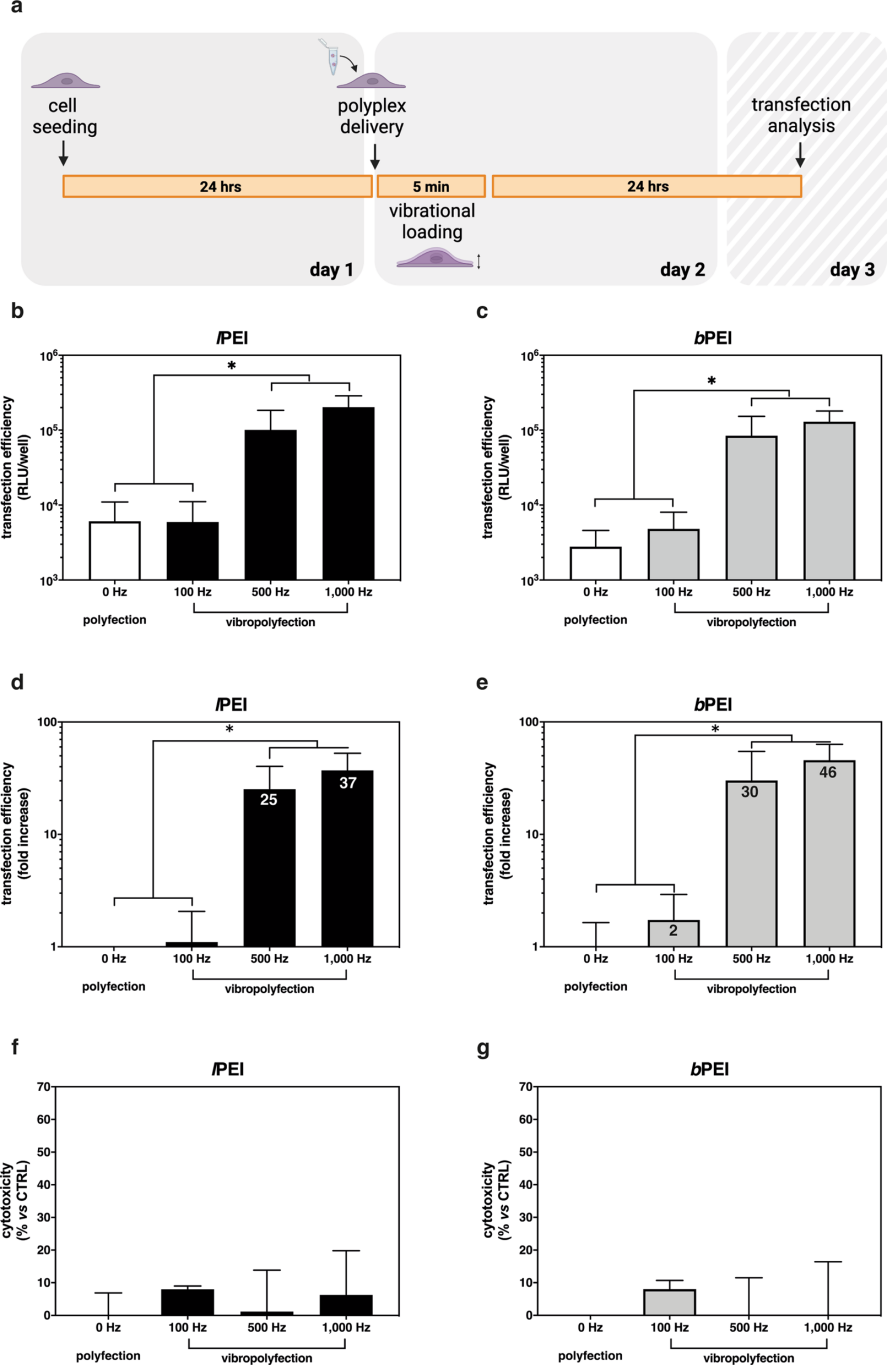
Comparative transfection efficiencies and cytotoxicities of *l*PEI/pDNA and *b*PEI/pDNA complexes delivered before mechanical cell stimulation (pre-delivery set-up). **a** Workflow of the pre-delivery vibropolyfection experimentation. Twenty-four hrs post-seeding, L929 cells were challenged with polyplexes, stimulated for 5 min at different frequencies (f_in_ = 100 Hz, 500 Hz, and 1,000 Hz), then cultured for a further 24 hrs in standard culture conditions. Cytotoxicity and transfection efficiency were assessed 24 hrs after adding polyplexes to the cells. The transfection efficiency is expressed as **b**, **d** *Gaussia* luciferase activity and **c**, **e** fold-increase in *Gaussia* luciferase activity of vibropolyfected over polyfected cells (standard transfections), while **f**, **g** cytotoxicity is the percent cell toxicity (%) as compared to unstimulated and untransfected cells (CTRL). **b**, **d**, and **f** refer to *l*PEI/pGLuc (black bars), while (**c**, **e**, and **g**) to *b*PEI/pGLuc (gray bars) complexes. Each number within bars in the panels **e** and **f** refers to the average fold increase value. Results are expressed as mean ± SD (n ≥ 4; *p < 0.05)

To explore whether the vibrational loading would promote endocytosis and consequent transfection, we challenged different cell types with non-viral gene delivery particles. For this purpose, PEI molecules (both *l*PEI and *b*PEI), *gold standard* polymeric transfectants [2, 15], were used to prepare *l*PEI/pDNA and *b*PEI/pDNA complexes at an amine-to-phosphate ratio (N/P) of 30, as previously described [46]. Polyplexes were dripped onto cells, which have next undergone a 5-min vibrational loading (vibropolyfection conditions; pre-delivery set-up) at f_in_ = 100 Hz, 500 Hz, and 1,000 Hz. Transfection efficiencies and cytotoxicities were evaluated 24 hrs post-cell stimulation (Fig. 4a), and results were compared to unstimulated (polyfected) control cultures.

It is apparent from Fig. 4b and c that, beyond the threshold of 100 Hz, the higher the f_in_ the higher the transfection efficiency. In this regard, vibropolyfection at f_in_ = 1,000 Hz with *l*PEI/pDNA (Fig. 4d) and *b*PEI/pDNA complexes (Fig. 4e) induced 37- and 46-fold increases in luciferase activity, respectively, as compared to polyfected cells. Besides, the cytotoxicity induced by vibropolyfection was from negligible to very mild as compared to unstimulated and untransfected controls (Fig. 4f, g), thus highlighting that the combination of vibrational loading and polyplexes does not affect cell viability. The above trends were confirmed by parallel experiments on other cell types (Additional file 1: Fig. S2) and through the delivery of another plasmid encoding the intracellular modified firefly luciferase (pGL3) (Additional file 1: Fig. S3). This is of utmost importance, as the transfection yields were not dependent on the type of pDNA delivered. Moreover, these results allowed us to discard any impact of the vibrational loading on the membrane properties, thus on the secretion of *Gaussia* luciferase.

Further, we questioned whether a short-lived vibrational loading would affect the general features of polyplexes (e.g., aggregation, sedimentation). It is apparent from Additional file 1: Fig. S4 that the hydrodynamic diameter (D_H_) and zeta-potential (ζ_P_) of *l*PEI/pDNA and *b*PEI/pDNA complexes that have been subjected to 5 min-vibrational loading at different frequencies were invariably similar to those of unstimulated polyplexes (p > 0.05) and stood at about 150 nm and + 20 mV, consistently with previous works [2, 46].

These results disclose that a short-lived vibrational loading induces abrupt cell membrane perturbations that markedly improve the effectiveness of non-viral gene delivery particles. As we found out that the higher the f_in_ applied to cells the higher the transfection efficiency of polyplexes, we purposely selected the f_in_ = 1,000 Hz to expand this work further. Indeed, at this frequency, either kind of polyplex reached its transfection maximum in terms of luciferase activity levels (Fig. 4b, c) and fold-changes over polyfection conditions (Fig. 4d, e).

Besides, and as expected, the delivery of the pristine pDNA (i.e., not complexed with any transfectant) to the cells gave rise to barely detectable transfection efficiencies (Additional file 1: Fig. S5). We hypothesized that the plasmalemma retained its physical integrity during and upon mechanical stimulation. To corroborate this finding, the microscopic inspection of the cell surface upon vibrational loading showed the absence of pores (Figs. 2a, 3a). In order to provide irrefutable evidence that the cell membrane integrity was intact upon vibrational loading, cells were mechanically stimulated for 5 min at f_in_ = 1,000 Hz in the presence of a membrane-impermeant dye. Trypan Blue (TB) staining made us exclude the presence of membrane pores in the cell membrane (Additional file 1: Fig. S6). This is a peculiarity of this kind of mechanical stimulation as compared to the most used physical methods for gene delivery. To the best of our knowledge, we are the first to report the mechano-modulation of transfection efficiency through a physical stimulus with no effect on the cell membrane integrity.

### Membrane remodeling is responsible for the increased transfection efficiency

**Fig. 5:**
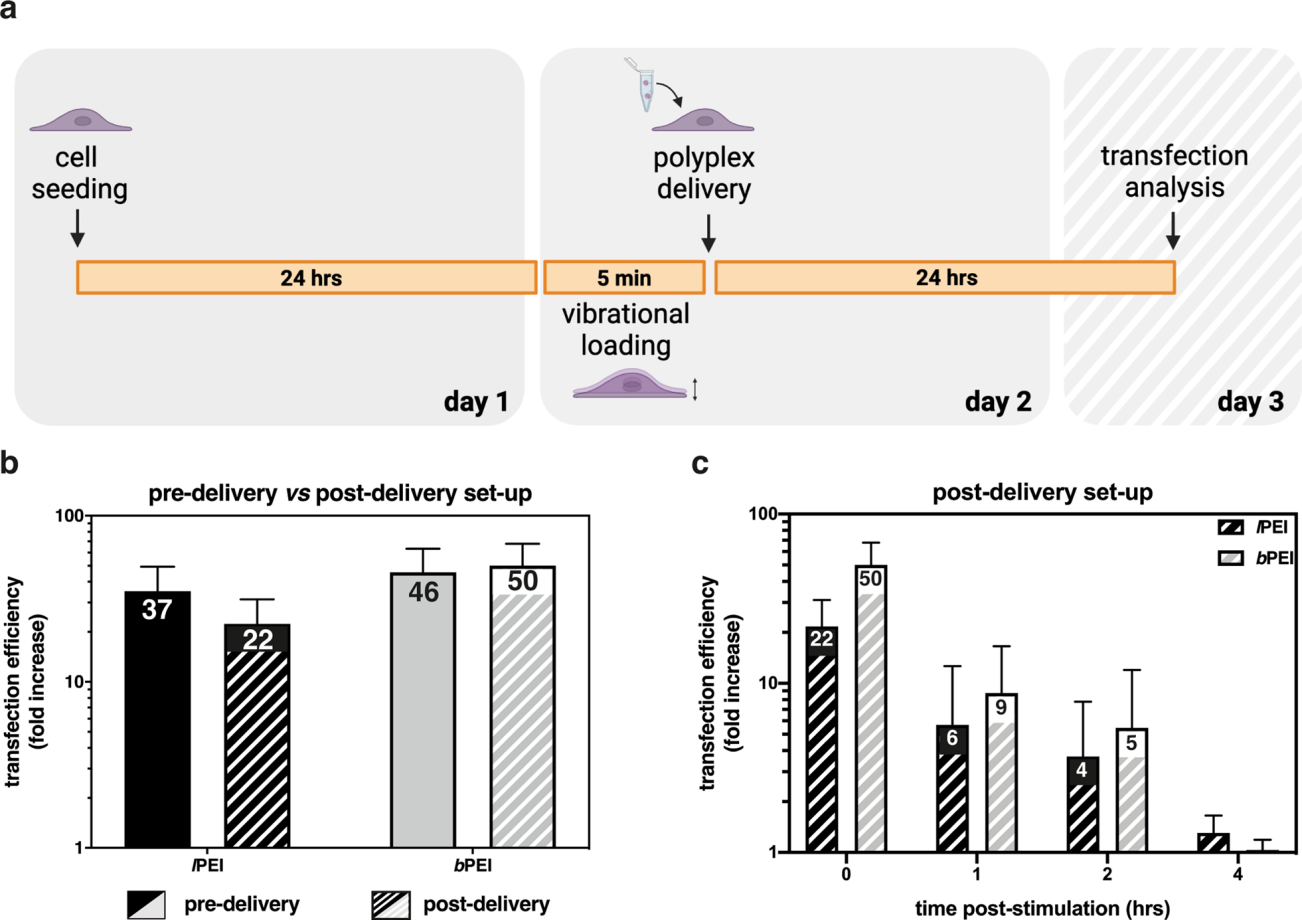
Comparative transfection efficiencies of *l*PEI/pDNA and *b*PEI/pDNA complexes delivered upon mechanical cell stimulation. **a** Workflow of the post-delivery vibropolyfection experimentation. Twenty-four hours post-seeding, L929 cells were stimulated for 5 min at f_in_ = 1,000 Hz, challenged with polyplexes, then grown for 24 hrs in standard culture conditions. Twenty-four hours after the addition of polyplexes to the cells, cytotoxicity and *Gaussia* luciferase activity were assessed. **b** Fold increase in *Gaussia* luciferase activity evaluated on L929 cells transfected with *l*PEI/pGLuc and *b*PEI/pGLuc following pre-delivery and post-delivery vibropolyfection protocols. **c** Fold increase in *Gaussia* luciferase activity evaluated on L929 cells transfected according to the protocol described in **a** at different periods (i.e., 0 hr, 1 hr, 2 hrs, and 4 hrs) from the discontinuation of the mechanical stimulation. Each number within bars in the panels **b** and **c** refers to the average fold-increase value of the dataset. Results are expressed as mean ± SD (n ≥ 3)

Transfections were purposely devised to shed further light on the cell mechanisms underpinning vibropolyfection. We compared the transgene expression of classically polyfected cells to that of vibropolyfected cells. Vibropolyfections were carried out according to two experimental set-ups we named pre-delivery (Fig. 4a) and post-delivery (Fig. 5a) depending on the timing of addition of polyplexes to the cells with respect to the application of the vibrational load (5 min-stimulation at f_in_ = 1,000 Hz).

Vibropolyfection invariably induced a significant increase in transfection levels of either kind of polymer as compared to standard polyfection (p < 0.05 vs. unstimulated cells) (Fig. 5b). These data point to long-lasting cell membrane recovery in response to short-lived plasmalemma perturbations, rather than cell membrane disruption itself, as the key mechanism responsible for the increased transfection efficiency observed in vibropolyfected cells.

In an attempt to gauge the time course of the cell response to the vibrational loading, cells were stimulated for 5 min at f_in_ = 1,000 Hz, and polyplexes were next added to cells at different times (i.e., 0 hr, 1 hr, 2 hrs, and 4 hrs) after the discontinuation of the mechanical stimulation, and transfection efficiency was evaluated after further 24 hrs.

We observed that the positive effect of the vibrational loading on transfection efficiency steadily decreased over time, alongside membrane recovery, and was practically lost 4 hrs upon the discontinuation of the stimulation (Fig. 5c). Nevertheless, 2 hrs post-stimulation, the transfection efficiencies of *l*PEI- and *b*PEI-based polyplexes were 3.7-fold and 5.4-fold higher than those of polyfected cells (static conditions), and the trend was similar for either kind of polyplex.

We can speculate that the cell membrane recovery that occurs in response to the mechano-induced plasmalemma blebbing and ruffling may favorably impact endocytosis [36, 56], which ultimately improves transfection efficiency. This mechanism is mechanistically and temporally distinct from those that lead to membrane poration [71–73], which may actually cause cell death because of the rapid ion influx/efflux [4].

Besides, this work is built on the assumption that vibrations are faithfully transmitted from the wave driver to the cells as these adhere to the culture vessel which, in turn, is integral to the driveshaft. To corroborate this, Jurkat cell suspensions underwent vibropolyfection, and transfection efficiencies were compared to polyfection. As expected, we found no effect of vibrational loading on cell transfection (Additional file 1: Fig. S7). This implies that the increase in transfection found in vibropolyfected cells really depends on the magnitude of the stimulus experienced, in sharp agreement with previous works [36].

### Vibrational loading enhances polyplex uptake

In order to uncover the reason(s) underpinning the mechano-mediated improvement of transfection shown in Fig. 5, we used quantitative real-time polymerase chain reaction (qRT-PCR) to compare the amount of pDNA internalized during classical transfection vs. vibropolyfection (post-delivery experimental set-up). We relied on the current knowledge that the cell behavior in response to membrane perturbations may result in the activation of membrane trafficking processes that give rise to the endocytic uptake of extracellular payloads [63, 74].

**Fig. 6:**
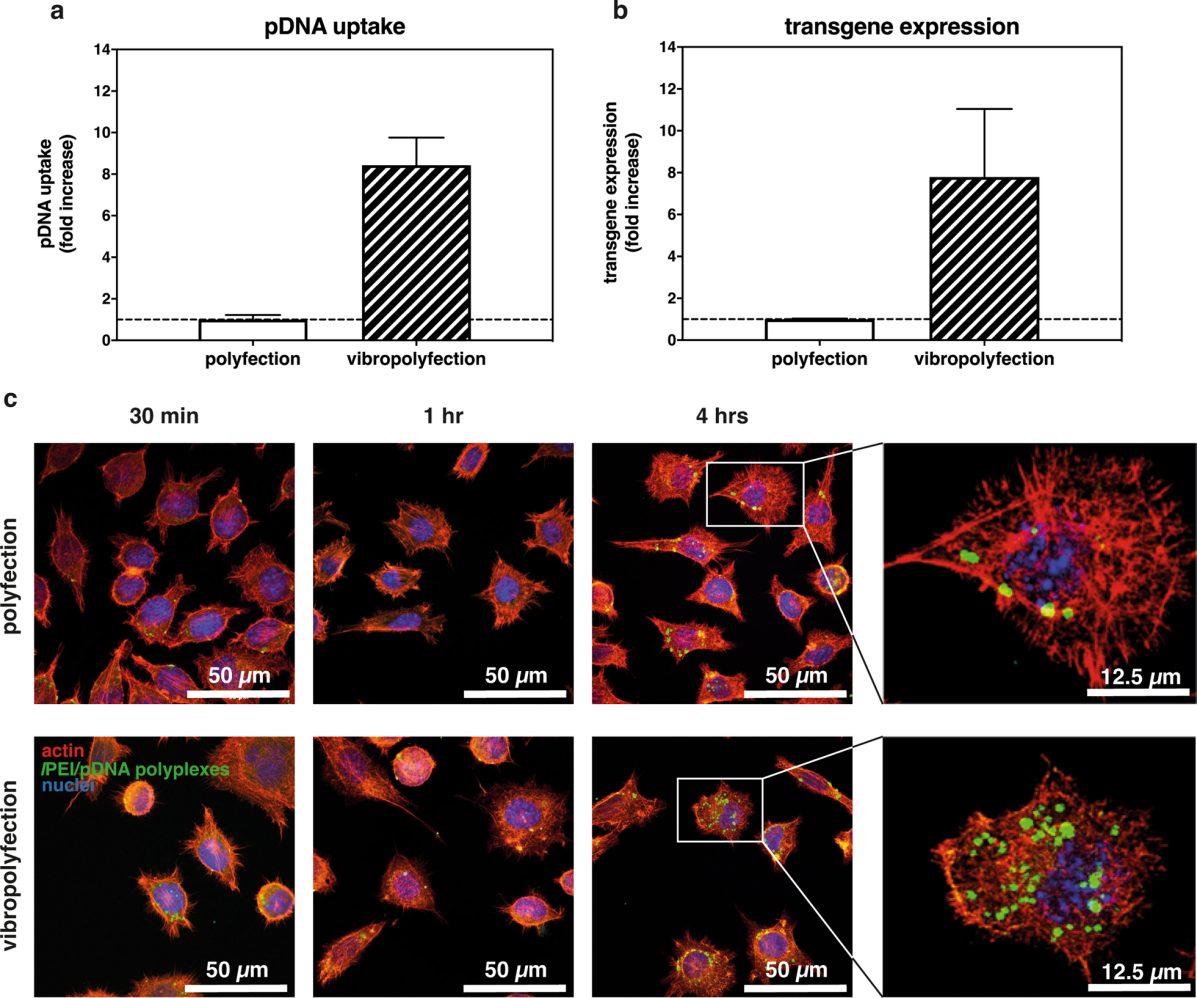
Effect of vibrational loading on polyplex uptake and transgene expression. **a** Fold-increase in pDNA uptake by polyfected vs. vibropolyfected L929 cells. Experiments were carried out with *l*PEI/pGLuc following the post-delivery set-up. Results express the fold-increase in the amount of intracellular pDNA (t = 24 hrs) in vibropolyfected cells with respect to polyfected counterparts. Results are expressed as mean ± SD (n = 3). **b** Expression levels of *Gaussia* luciferase transcripts in L929 cells polyfected and vibropolyfected with *l*PEI/pGLuc. Target luciferase transcript levels were normalized to GAPDH levels. Results are expressed as mean ± SD (n = 3). **c** Uptake kinetics of *l*PEI/pDNA-FITC polyplexes. CLSM images of L929 cells transfected according to standard transfection vs. vibropolyfection (post-delivery set-up, 1,000 Hz-vibrational loading for 5 min). Cells were next fixed and imaged at 30 min, 1 hr, and 4 hrs post-polyplex addition to the cells. High-magnification (insets of **c**, right panels) digital images of single cells were taken 4 hrs after polyfection or vibropolyfection (post-delivery set-up, 1,000 Hz-vibrational loading for 5 min). Polyplexes are in green, while F-actin and nuclei are in red and blue, respectively. Scale bars = 50 µm and 12.5 µm (right panels)

It turned out that only ≈ 1.5% of the initial pDNA delivered through polyfection was internalized by cells (≈ 9.5 × 10^3^ pDNA copies internalized/cell *vs.* theoretical 6.3 × 10^5^ pDNA copies delivered/cell), in good agreement with literature data [75]. Conversely, the pDNA uptaken by vibropolyfected cells was ≈ 13% (≈ 8 × 10^4^ pDNA copies/cell), which means almost a log increase in NAs internalization in mechanically-loaded cells (Fig. 6a). We assumed that the boost in transfection efficiency found in vibropolyfected cells (Fig. 5) was due to the increased internalization of polyplexes as a result of the membrane recovery. Because only a small proportion of the already little portion of pDNA that is internalized reaches the nucleus and is transcribed actually [76, 77], we gauged the transgene expression in polyfected and vibropolyfected L929 cells and compared them. In line with the internalization findings, vibropolyfection induced an ≈ 8  fold-increase in *Gaussia* luciferase transcripts, as compared to polyfection (Fig. 6b), meaning that vibropolyfection capitalizes on boosting the endocytosis of polyplexes to improve the transfection efficiency.

To shed some light on the mechano-induced events that increase the uptake of polyplexes, we used confocal laser scanning microscopy (CLSM) and track into cells fluorescently labeled polyplexes upon vibropolyfection and classical polyfection. It is apparent that the number of internalized *l*PEI/pDNA-FITC complexes invariably increased over a 4-hrs time lag **(**Fig. 6c). Nevertheless, the number of polyplexes in vibropolyfected cells was significantly greater than in classically transfected cells. Imaging at higher magnification (Fig. 6c, rightmost panels) allows localizing polyplexes in single cells. As expected, there were fewer polyplexes in the cytosol of cells transfected under standard conditions, and some pDNA was localized close to or co-localized with the nucleus of vibropolyfected cells.

These findings strongly support the hypothesis that the vibrational loading triggers mechano-induced cell membrane remodeling. This results in an increased uptake of polyplexes that, in turn, gives rise to considerably high transfection efficiency.

### Mechanically triggered clathrin-mediated endocytosis is responsible for enhanced polyplex uptake

**Fig. 7:**
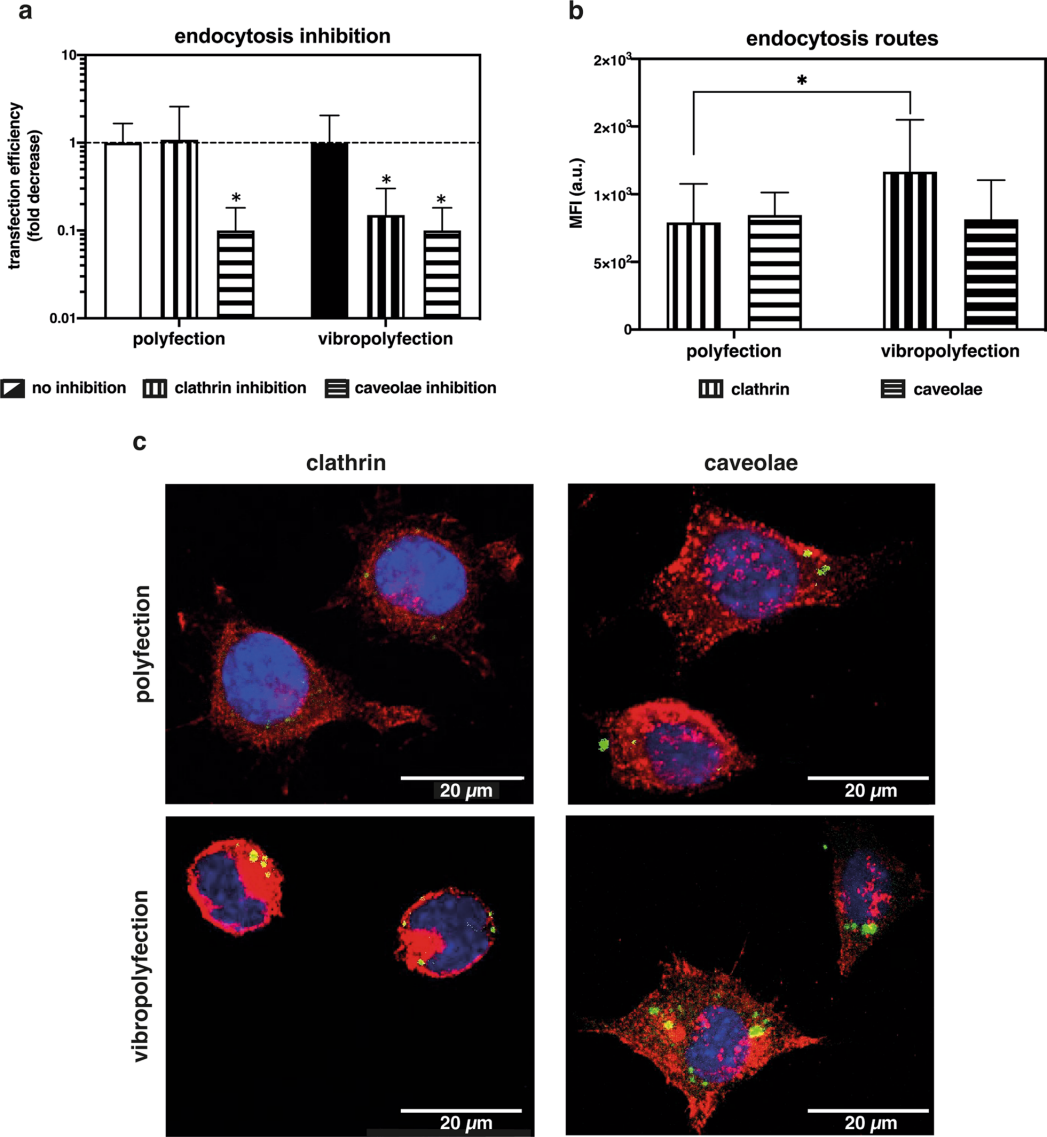
Effect of vibrational loading on clathrin- and caveolae-mediated endocytosis and transfection. **a** Effect of clathrin- and caveolae-mediated endocytosis inhibitors on polyfected and vibropolyfected (5 min, f_in_ = 1,000 Hz, post-delivery set-up) L929 cells. Briefly, cells were pre-treated for 30 min with chlorpromazine (10 µg/mL) or filipin (5 µg/mL) to inhibit clathrin- and caveolae-mediated endocytosis, respectively. Cells were next challenged with *l*PEI/pGL3 polyplexes for 3.5 hrs and cultured for a further 20 hrs. Data are expressed as a fold decrease in transfection efficiency in cells treated with inhibitors compared to cells transfected in the absence of inhibitors. Data are expressed as mean ± SD (n = 3, * p < 0.05 vs. cells transfected in the absence of inhibitors). **b** Effect of vibrational loading on clathrin and caveolae expression in polyfected and vibropolyfected L929 cells. Mean Fluorescence Intensity (MFI) of clathrin heavy chain and caveolin-1 proteins normalized to the cell area after transfection with *l*PEI/pDNA-FITC complexes upon static (polyfection) and vibropolyfection conditions (post-delivery set-up, f_in_ = 1,000 Hz). Data are expressed as mean ± SD (n = 3, * p < 0.05). **c** Representative CLSM images of polyfected and vibropolyfected (post-delivery set-up, f_in_ = 1,000 Hz) L929 cells. Briefly, cells were transfected for 1 hr, fixed, and clathrin heavy chain and caveolin-1 were immunostained. *l*PEI/pDNA-FITC polyplexes are in green, clathrin heavy chain and caveolin-1 are in red, while nuclei are in blue. Scale bar = 20 µm

There is a general consensus that two distinct endocytic pathways may contribute to polyplex internalization [78, 79]. In our study, L929 cells were treated with specific inhibitors of clathrin- and caveolae-dependent endocytosis, named chlorpromazine and filipin, respectively, prior to polyfection and vibropolyfection (post-delivery set-up). Chlorpromazine (10 µg/mL) and filipin (5 µg/mL) were used at a dose causing little-to-mild cytotoxicity (Additional file 1: Fig. S8), such that the sole effect of internalization blockade on the transfection efficiency could be determined. In classically polyfected cells, transfection efficiency was not affected by the inhibition of clathrin-mediated endocytosis (Fig. 7a), whereas luciferase expression was dramatically reduced by caveolae inhibition. These results are not surprising, given that PEI-based polyplexes are mostly internalized via caveolae [78, 80]. Instead, in vibropolyfected cells, the separate inhibition of clathrin- and caveolae-mediated endocytosis significantly impaired transfection efficiency (Fig. 7a). These results indicate that the vibrational loading triggers the activation of clathrin- and caveolae-mediated endocytic pathways. Such behavior relies heavily upon mechano-chemical feedback operated by cells in response to changes in membrane tension, which ultimately impacts active transport pathways [74, 81]. In line with this observation, it was shown that changes in membrane tension caused by the application of external stimuli to cells result in an upregulation of clathrin-mediated endocytosis [56, 81]. In this context, caveolae likely account for rapid compensation to the abrupt changes in membrane tension, while other endocytosis mechanisms and actin remodeling take longer to initiate [74, 82].

To gain more insights into the role of clathrin- and caveolae-mediated endocytosis on polyplex uptake, CLSM analyses were performed on cells transfected via polyfection vs. vibropolyfection. One hr after the addition of polyplexes to cells, clathrin heavy chain and caveolin-1 proteins were immunostained. Interestingly, vibropolyfected cells showed significantly higher clathrin levels with respect to classically polyfected cells (p < 0.05) (Fig. 7b). On the other hand, there was no difference in caveolae levels between the two conditions, as also qualitatively shown in Fig. 7c.

Overall, these findings support the hypothesis that a short-lived vibrational loading induced increased cell uptake of polyplexes as a result of significant membrane remodeling.

## Conclusions

We developed a safe, user-friendly, and efficient in vitro transfection strategy relying on the short-lived mechanical stimulation of cells undergoing polyfection. The vibrational load was very gentle to cells, ensuring no impact on cell viability and gross phenotype. Conversely, cell priming with a high-frequency short-lived mechanical stimulation caused a dramatic increase in clathrin-mediated polyplex uptake as a result of reversible rearrangements of the cell membrane. Accordingly, there was a remarkable improvement in transfection efficiency when using either kind of PEI-based polyplex on different kinds of adherent cell types. Future work will also address the effect of vibrational loading on the transfection behavior of lipoplexes, which are typically internalized by cells through clathrin-mediated endocytosis [80, 83, 84]. In conclusion, this study paves the way for using mechanical cell stimulation(s) to improve the effectiveness of non-viral gene delivery vectors and develop ever more reliable in vitro and ex vivo gene delivery approaches. Further research will allow gaining breadth and depth in the underlying mechanisms of membrane remodeling and the consequent activation of mechano-sensitive endocytic pathways, and figure out whether this cell stimulation could improve the effectiveness of other gene delivery vectors and different kinds and sizes of NAs.

## Methods

### Materials

pGL3 (pDNA encoding the modified Firefly luciferase, pGL3-Control Vector, 5,256 bp) and Luciferase Assay System were obtained from Promega (Milan, Italy), while pGLuc (pDNA encoding the secreted *Gaussia* Luciferase, pCMV-GLuc 2 Control Plasmid, 5,802 bp) and BioLux^®^ Gaussia Luciferase Assay Kit were purchased from New England BioLabs (Ipswich, MA, USA). pGL3 and pGLuc were amplified, isolated, purified, and diluted in 0.1 × TE buffer (1 mM Tris, pH 8; 0.1 mM EDTA) as previously described [85].

25 kDa *l*PEI (cat. nr. 23966) was from Polysciences (Eppelheim, Germany), 25 kDa *b*PEI (cat. nr. 40872-7) was from Merck Life Science (Milan, Italy). L929 (murine fibroblasts from subcutaneous connective tissue, CCL-1), HeLa (human ovarian carcinoma epithelial cells, CCL-2), MG-63 (human osteosarcoma fibroblasts, CRL-1427), and Jurkat (T lymphoblasts, TIB-152) cell lines were purchased from the American Type Culture Collection (ATCC^®^, Manassas, VA, USA), while bAPCs were isolated from metacarpophalangeal joints of 8-month-old bovines [86]. Bicinchoninic Acid (BCA) Protein Assay Kit was purchased by ThermoFisher Scientific (Monza, Italy) while Alamar Blue Assay® was from Life Technologies (Monza, Italy). Fast-Tag Basic Labeling Kit was from Vector Laboratories (Burlingame, CA, USA). Hoechst 33342, mouse anti-caveolin 1 monoclonal antibody (MA3-600), and mouse anti-clathrin heavy chain monoclonal antibody (MA1-065) were from Thermo Fisher Scientific. All the other reagents were from Merck Life Science unless otherwise specified.

### Vibrational loading device

The vibrational loading device (Fig. 1a) comprises (i) a sine wave signal generator (Lascells, Market Drayton, UK), able to modulate the wave frequencies from 0.1 Hz to 10,000 Hz and characterized by a peak-to-peak wave amplitude from 0 to 24 V, connected to (ii) a mechanical wave driver (Arbor Scientific, Ann Arbor, MI, USA), equipped with (iii) a driveshaft moving in vertical (z-axis) direction. For dynamic transfection assays, multi-well plates were placed onto (iv) a built-in-home cell culture plate housing in order to make the plate jointly settled to the driveshaft (Fig. 1a).

The shaft displacement along the z-axis was assessed as a function of the wave frequency. To do so, a *homemade* accelerometer sensor was mounted over the plate housing and accelerations at different wave frequencies (i.e., 10 Hz, 50 Hz, 100 Hz, 200 Hz, 300 Hz, 400 Hz, 500 Hz, 600 Hz, 700 Hz, 800 Hz, 900 Hz, and 1,000 Hz) were acquired. Raw data in the time domain were then processed using a custom code in Matlab software (https://it.mathworks.com/products/matlab.html) and transformed using a FTT technique to identify the dominant frequency. Displacement values, expressed in terms of s(t), were calculated from accelerations data as follows (Eq. 1):

1 $$s\left(t\right)= \frac{{A}_{p}}{{\omega }^{2}}=\frac{{A}_{p}}{{(2\pi f)}^{2}}$$

where *A*_*p*_ is the peak value of the acceleration, ω is the angular frequency (expressed as $\omega =2\pi f$) and *f* is the sine wave frequency.

### Cell culture

Mycoplasma-free adherent L929, HeLa, MG-63 cells, and bAPCs were cultured in Dulbecco’s Modified Eagle’s Medium (DMEM), supplemented with 10% (v/v) fetal bovine serum (FBS), 1 mM sodium pyruvate, 10 mM HEPES, 100 U/mL penicillin, 0.1 mg/mL streptomycin and 2 mM glutamine (hereinafter referred to as complete DMEM, cDMEM).

Mycoplasma-free Jurkat cells were cultured in Roswell Park Memorial Institute (RPMI) 1640 medium, supplemented with 10% FBS, 100 U/mL penicillin, and 0.1 mg/mL streptomycin.

Cells were cultured at 37 °C in a humidified atmosphere under constant supply of 5% (v/v) CO_2_ (hereafter referred to as standard culture conditions).

### Evaluation of vibrational loading on cell viability

To assess the effect of vibrational loading on cell viability, adherent cells were seeded onto  96-well plates at a density of 2 × 10^4^ cell/cm^2^ and maintained in standard culture conditions for 24 hrs. Afterward, cells were stimulated for 5 min at different vibration frequencies (f_in_ = 10 Hz, 50 Hz, 100 Hz, 500 Hz, and 1,000 Hz) and maintained in standard culture conditions. Twenty-four hrs after the discontinuation of the stimulation, cell viability was evaluated using AlamarBlue^®^ assay according to the manufacturer’s instructions. Briefly, the medium was removed from each well and replaced with 100 µL/well of 1 × resazurin dye solution in cDMEM. Next, plates were incubated in standard culture conditions for 2 hrs, then the fluorescence was read with a Synergy H1 reader (BioTek, Winooski, VT, USA) (λ_ex_ = 540 nm; λ_em_ = 595 nm). The viability of unstimulated cells (CTRL) was assigned to 100% and the viability of stimulated ones was determined according to Eq. 2:

2 $$\mathrm{Viability }\,[\mathrm{\%}]=\frac{{\text{F}}_{\text{stimulated cells}}}{{\text{F}}_{\text{CTRL}}} \times 100$$

where F is the recorded fluorescence.

### Evaluation of vibrational loading on cell proliferation rate

To evaluate the role of vibrational loading on cell proliferation rate, L929 cells were seeded onto 96-well plates at a density of 2 × 10^4^ cell/cm^2^ and maintained in standard culture conditions for 24 hrs. At 24 hrs post-seeding, AlamarBlue^®^ viability assay was carried out as described above. Next, the medium was replaced with 100 µL/well of fresh cDMEM, and cells were stimulated for 5 min at different vibration frequencies (f_in_ = 10 Hz, 50 Hz, 100 Hz, 500 Hz, and 1,000 Hz) and maintained in standard culture conditions for further 24 hrs. Forty-eight hrs post-seeding, the viability assay was repeated as described above. Cell doubling time was then calculated as follows (Eq. 3):

3 $$\mathrm{Doubling\, time }[\mathrm{hr}] =\mathrm{ log}2(\frac{{\text{F}}_{\text{t48}}}{{\text{F}}_{\text{t24}}})$$

where F_t48_ and F_t24_ are the fluorescence signals recorded at 48 and 24 hrs post-seeding, respectively.

### Evaluation of vibrational loading on cell morphology

To explore the effects of the vibrational loading on cell morphology, cells were observed using a StereoScan 360 SEM (Cambridge Instruments, London, UK). Briefly, cells (L929, HeLa, MG-63 cells, and bAPCs) were seeded at a density of 2 × 10^4^ cell/cm^2^ on sterile glass coverslips (Ø = 15 mm) inserted into separate wells of 24-well plates, and cultured in standard culture conditions for 24 hrs. Twenty-four hrs post-seeding, cells were stimulated for 5 min at different vibration frequencies (f_in_ = 10 Hz, 50 Hz, 100 Hz, 500 Hz, and 1,000 Hz). Cells were next fixed right after the stimulation was discontinued or at different time periods post-stimulation (i.e., 5 min, 30 min, and 60 min). Unstimulated cells were used as controls. For SEM imaging, cells were fixed in 2% (v/v) glutaraldehyde solution for 20 min, dehydrated with graded ethanol series, then samples were gold-sputtered, mounted onto stubs, and examined using an accelerating voltage of 10 kV. SEM images were acquired at ×1,500 and ×7,000 magnification.

The size of membrane outgrowths was measured using ImageJ software. Briefly, 10 blebs per cell were contoured with circles in order to compute the average diameter. Three images per stimulation frequency per cell type were analyzed.

### Evaluation of vibrational loading on cell membrane permeability

To evaluate the role of vibrational loading on cell membrane permeability, L929 cells were seeded onto 96-well plates at a density of 2 × 10^4^ cell/cm^2^ and maintained in standard culture conditions for 24 hrs. At 24 hrs post-seeding, the medium was removed, and cells were incubated for 5 min with 100 µL/well of 0.4% (w/v) solution of TB in cDMEM (1:1 (v/v)) and kept in either static conditions or stimulated for 5 min at 1,000 Hz. Next, cells were washed with PBS, detached from wells by using 1 × trypsin–EDTA solution in PBS, and counted using a cell counting chamber. As positive permeabilization control, cells were incubated with 0.01% (v/v) Triton X-100 solution for 1 min before incubation with the TB solution. The number of TB-positive cells was calculated as a percentage with respect to the total cell number as follows (Eq. 4):

4 $$\mathrm{TB}-\mathrm{positive \,cells }(\mathrm{\%}) = \frac{{N}_{TB-positive cells}}{{N}_{live cells}+ {N}_{TB-positive cells}}\times 100$$

### Evaluation of vibrational loading on polyplex behavior

#### Polyplex preparation

25 kDa *l*PEI and 25 kDa *b*PEI were diluted in 10 mM HEPES to reach a final polymer concentration of 0.86 mg/mL, corresponding to an amine concentration ([N]) of 20 mM, considering that there is one nitrogen per repeat PEI unit (–NHCH_2_CH_2_–, Mw = 43 Da) [2].

All reagents were pre-warmed at room temperature (r.t.) prior to use. Polyplexes were prepared at r.t. by adding the aqueous solution of pDNA (0.25 µg/µL) to PEI solutions at a stoichiometric ratio of 1:10 (v/v) to give a final DNA concentration of 20 ng/µL and N/P 30 [46], where N/P is defined as the number of amines (N) of the PEIs used to complex the phosphate groups (P) of a given amount of pDNA. PEI/pDNA polyplexes were incubated for 20 min at r.t., then used in further experiments.

#### Physicochemical characterization of polyplexes

To evaluate polyplex stability upon vibrational loading, their physicochemical characteristics, namely size (in terms of D_H_) and the overall surface charge (in terms of ζ_P_), were evaluated. Briefly, 50 µL of PEI-based complexes containing 1 µg of pDNA prepared as described here above were transferred in separate wells of a 96-multiwell plate and challenged with vibrational loading for 5 min at 100 Hz, 500 Hz, and 1,000 Hz. Unstimulated polyplexes were used as controls. Afterwards, polyplexes were diluted 1:9 (v/v) in 10 mM HEPES, then the D_H_ and the ζ_P_ were evaluated at r.t. by Dynamic Light Scattering (DLS) and Electrophoretic Light Scattering (ELS), respectively, using a Malvern Zetasizer Nano ZS instrument (Malvern, UK), fitted with a 5 mV HeNe laser (λ = 633 nm) and a scattering angle of 173°.

### Evaluation of the effects of vibrational loading on transfection efficiency

#### In vitro cell transfection assays

Transfection assays were performed on L929, HeLa, MG-63, bAPCs, and Jurkat cells. Briefly, L929, HeLa, MG-63 cells, and bAPCs were seeded onto 96-well plates at a density of 2 × 10^4^ cell/cm^2^ and maintained in standard culture conditions for 24 hrs, while Jurkat cells were plated at 10^6^ cell/mL in 200 µL/well onto 48-well plates just before transfection. For transfection assays, fresh polyplexes were prepared (N/P 30) as described here above by complexing 160 ng/cm^2^ of pDNA (or 320 ng/cm^2^ for transfecting bAPCs and Jurkat cells) with PEI solutions. Transfection assays were carried out either in standard (i.e., static) conditions or upon vibrational loading (vibropolyfection conditions).

For standard conditions (polyfection), cells were challenged with 100 µL/well of polyplexes-containing culture medium (or naked pDNA-containing medium), then incubated under standard culture conditions for 24 hrs. These cells served as internal controls for transfection.

For vibropolyfection, 2 different experimental set-ups were employed: (i) pre-delivery set-up (Fig. 4a): cells were challenged with polyplexes, then immediately stimulated for 5 min at different frequencies (100 Hz, 500 Hz, and 1,000 Hz). Following 5 min-stimulation, cells were cultured under standard conditions for 24 hrs; (ii) post-delivery set-up (Fig. 5a): cells were stimulated for 5 min at 1,000 Hz, immediately challenged with polyplexes (or naked pDNA), then cells were cultured in standard culture conditions for 24 hrs.

#### Evaluation of cytotoxicity

Twenty-four hrs after the addition of the polyplexes to the cells, the cytotoxicity was evaluated using the AlamarBlue^®^ viability assay according to the manufacturer’s instructions. Briefly, the medium was removed from each well and replaced with 100 µL/well of 1 × resazurin dye solution in cDMEM. Next, the plates were incubated in standard culture conditions for 2 hrs, then the fluorescence was read with a Synergy H1 reader (λ_ex_ = 540 nm; λ_em_ = 595 nm). The viability of unstimulated, untransfected cells (CTRL) was assigned to 100% and the cytotoxicity was determined as follows (Eq. 5):

5 $$\text{Cytotoxicity} \,[\%] = 100 \% - \text{Viability}\, [\%] = \left(1 - \frac{{\text{F}}_{\text{transfected cells}}}{{\text{F}}_{\text{CTRL}}}\right) \times 100$$

where F is the recorded fluorescence.

#### Evaluation of transfection efficiency

Transgene expression was evaluated 24 hrs after polyplexes delivery by measuring the luciferase activity in the culture media (secreted *Gaussia* luciferase) or cell lysates (intracellular firefly luciferase), depending on the plasmid used (pGLuc and pGL3, respectively).

To evaluate the overall luciferase activity, 20 µL of either cell supernatant (for pGLuc-transfected cells) or cell lysate (for pGL3-transfected cells) were mixed with 50 µL of the corresponding luciferase assay substrate. The luminescence signal (expressed as Relative Light Units, RLU) was measured by means of a Synergy H1 reader.

When pGLuc was used, the transfection efficiency was simply related to the *Gaussia* luciferase activity expressed as RLU/well, while when pGL3 was employed, firefly luciferase signals were normalized to the total protein content determined by BCA assay, and transfection efficiency was expressed as RLU/mg of proteins.

For each vibropolyfection condition, the fold-increase in transfection efficiency with respect to (static) polyfection was calculated as well.

#### Quantification of plasmid internalization

Plasmid uptake was quantified in transfected L929 cells via qRT-PCR. Briefly, 24 hrs after *l*PEI/pGLuc polyplexes delivery either via classical polyfection or vibropolyfection (post-delivery set-up), cells were washed with sterile PBS and total DNA from whole cells was extracted with SingleShot Cell Lysis kit (24.5 µL/well: 24 µL/well of SingleShot Cell Lysis and 0.5 µL/well of Proteinase K solution; cat. no. 172581, BioRad Laboratories, Segrate, Italy), according to the manufacturer’s instructions. Samples were then diluted 1:10 (v/v) in DNAse-free dH_2_O and the pGLuc sequence was amplified using the 5′-GGGTGGACTATTTACGGTAAACTGC-3′ forward primer and the 5′-TCAGAAGCCATAGAGCCCACCGCAT-3′ reverse primer. RT-PCR reactions were carried out in a CFX Connect Real-Time PCR Detection System (Bio-Rad Laboratories) using the SsoAdvancedTM Universal SYBR^®^ Green Supermix (BioRad Laboratories) (hereafter referred to as the master mix) as recommended by the manufacturer. Briefly, 10 µL of the reaction mix, containing 5 µL of 2 × master mix, 1.6 µL of 6 × primers solutions (final concentration: 200 nM per primer), 2.4 µL of DNAse-free dH_2_O, and 1 µL of DNA sample, were subjected to the following thermocycling conditions: 95 °C for 2 min (polymerase activation); 95 °C for 15 s (DNA denaturation) followed by the annealing/extension step performed at 60 °C for 90 s (40 PCR cycles). qRT-PCR assays were run in triplicate. For each sample, a melt-curve analysis (65–95 °C, 0.5 °C steps) was also performed to assess the specificity of the template amplification.

Absolute quantification of pGLuc in transfected cells was achieved by comparing the C_T_ values of test samples to a standard curve generated by ten-fold serial dilution of the pGLuc template. Data were expressed as a fold-increase in pDNA uptake in vibropolyfected cells with respect to the polyfected counterparts.

#### Quantification of transgene expression

Luciferase gene expression was assessed and compared in transfected L929 cells. Briefly, 24 hrs after *l*PEI/pGLuc polyplexes addition and following polyfection or vibropolyfection (post-delivery set-up), cells were washed with sterile PBS and total RNA from whole cells was extracted with SingleShot Cell Lysis kit (25 µL/well: 24 µL/well of SingleShot Cell Lysis, 0.5 µL/well of DNase solution and 0.5 µL/well of Proteinase K solution; BioRad Laboratories), according to the manufacturer’s instructions. RT-PCR reactions were carried out in a CFX Connect Real-Time PCR Detection System using the Cells-to-C_T_ 1-Step TaqMan^®^ Kit (Invitrogen^TM^, ThermoFisher Scientific) as recommended by the manufacturer. Briefly, 10 µL of reaction mix, containing 2.5 µL of TaqMan^®^ 1-Step qRT-PCR Mix, 0.5 µL of 20 × TaqMan^®^ Gene expression Assay targeting GAPDH or luciferase, 6 µL of DNAse-free dH_2_O and 1 µL of mRNA sample, were subjected to the following conditions: 1 cycle at 50 °C for 5 min (reverse transcription, RT), 95 °C for 20 s (reverse transcriptase inactivation), 95 °C for 15 s (denaturation) followed by annealing/extension step performed at 60 °C for 60 s (40 PCR cycles). RT-PCR assays were run in triplicate. GAPDH was used as the housekeeping gene and the relative quantification of luciferase mRNA levels (fold-change with respect to GAPDH in each sample) were calculated using the 2^−ΔΔCt^ method.

#### Evaluation of polyplex uptake through CLSM

CLSM analyses were carried out to shed light on the role of mechanical stimulation on polyplex uptake. First, pGLuc was covalently labeled with FITC by means of the Fast-Tag Basic Labelling kit (MB-8000, Vector Laboratories) according to the manufacturer’s instructions. L929 cells were seeded at 2 × 10^4^ cell/cm^2^ onto sterile glass coverslips (Ø = 15 mm), which were next inserted into separate wells of 24-well plates and cultured in standard culture conditions for 24 hrs. Twenty-four hrs post-seeding, cells were challenged with *l*PEI/pDNA-FITC polyplexes (N/P 30) according to the vibropolyfection protocol (post-delivery set-up). Unstimulated polyfected samples were used as controls. At given time points, namely 30 min, 1 hr, and 4 hrs after polyplex addition to cells, coverslips were rinsed with PBS, fixed in 4% (w/v) paraformaldehyde for 20 min, and permeabilized for 30 min at r.t. with a blocking solution (0.1% (v/v) Triton X-100, 3% (w/v) BSA in PBS).

To better localize *l*PEI/pGLuc-FITC complexes within the cells, cellular F-actin was stained with TRITC-Rhodamine Phalloidin (1:25 (v/v) in blocking solution) (ThermoFisher Scientific), while nuclei were counterstained with Hoechst 33342 (1:1,000) (ThermoFisher Scientific). Stained samples were subsequently mounted on glass slides and digital images were taken with a Nikon A1 Confocal Microscope.

#### Evaluation of polyplex uptake by endocytosis inhibition and protein expression

Transfection assays in the presence of endocytosis inhibitors were carried out to investigate the contribution of each endocytic route on the polyplex uptake by cells undergoing polyfection and vibropolyfection. Briefly, L929 cells were seeded onto 96-well plates at a density of 2 × 10^4^ cell/cm^2^ and cultured in standard culture conditions for 24 hrs. Twenty-four hrs post-seeding, cells were pre-treated with subtoxic concentrations of either chlorpromazine (10 µg/mL) or filipin (5 µg/mL) (please refer to Additional file 1: Fig. S8). The medium was replaced and the cells either underwent vibrational loading at 1,000 Hz for 5 min or were rested. Cells were next challenged with *l*PEI-based polyplexes for 3.5 hrs, the medium was replaced, and cells were cultured in standard conditions for 20 hrs before assessing the cytotoxicity and the transfection efficiency. Transfected cells in the absence of inhibitors served as positive controls.

Immunostaining of clathrin and caveolae was performed as well. Briefly, L929 cells were seeded at 2 × 10^4^ cell/cm^2^ onto sterile glass coverslips (Ø = 15 mm) and transfected for 1 hr as described above. Following fixation and permeabilization, samples were incubated overnight at 4 °C with mouse anti-clathrin heavy chain (1:500) and mouse anti-caveolin 1 (1:500) primary antibodies. After extensively washing with 0.01% (v/v) Tween-20 in PBS, the samples were incubated for 1 hr at r.t. in the dark with Cy3-labelled rabbit anti-mouse (1:300) secondary antibody. Cell nuclei were counterstained with Hoechst 33342 (1:1,000).

Immunostained samples were mounted on glass slides and digital images were taken with Nikon A1 Confocal Microscope. The mean fluorescence intensity of clathrin and caveolae in test samples was calculated with JACoP plugin, under ImageJ software. Fifty images taken from three different replicates were analyzed for each condition.

### Statistical analysis

Statistical analyses were carried out using Prism version 8 (GraphPad software, La Jolla, CA). All data collected from at least three independent experiments were initially analyzed using the D'Agostino & Pearson omnibus normality test. Unpaired t-test and one-way ANOVA (multiple comparisons) with posthoc Tukey test were used to compare two or more experimental groups, respectively. Significance was retained when p < 0.05. Data are expressed as mean ± standard deviation (SD, n ≥ 3).

## Supplementary Information

**Additional file 1: Figure S1.** Effect of vibrational loading on the viability and morphology of HeLa and MG-63 cells, and bAPCs. **Table S1.** Evaluation of bleb size in vibrational-loaded cells. **Figure S2.** Effect of the cell stimulation on the transfection efficiency and cytotoxicity of lPEI/pGL3 and bPEI/pGL3 complexes. **Figure S3.** Comparative transfection efficiencies of lPEI/pGL3 and bPEI/pGL3 complexes delivered before mechanical cell stimulation. **Figure S4.** Effect of vibrational loading on the physicochemical features of polyplexes. **Figure S5.** Evaluation of the effect of vibrational loading on naked pDNA delivery. **Figure S6.** Effect of vibrational loading on cell membrane permeability. **Figure S7.** Effect of the vibrational loading on the cytotoxicity and transfection efficiency of lPEI/pGL3 and bPEI/pGL3 polyplexes on Jurkat cell suspensions. **Figure S8.** Effect of two endocytosis inhibitors on the viability of polyfected and vibropolyfected L929 cells.

## Data Availability

All data generated or analyzed during this study are included in this published article and in its supplementary information files. The datasets analyzed during the current study are available from the corresponding author on reasonable request.

## Abbreviations

NAs: Nucleic acids
*l*PEI: Linear polyethyleneimine
*b*PEI: Branched polyethyleneimine
FFT: Fast Fourier Transformation
DNA: Deoxyribonucleic acid
bAPCs: Bovine articular primary chondrocytes
SEM: Scanning Electron Microscopy
qRT-PCR: Quantitative Real-Time Polymerase Chain Reaction
CLSM: Confocal Laser Scanning Microscopy
FITC: Fluorescein Isothiocyanate
BCA: Bicinchoninic Acid
RLU: Relative Light Units
RFU: Relative Fluorescence Units
MFI: Mean Fluorescence Intensity
DMEM: Dulbecco’s Modified Eagle’s Medium
TB: Trypan Blue
HEPES: 4-(2-Hydroxyethyl)-1-piperazineethanesulfonic acid
DLS: Dynamic Light Scattering
ELS: Electrophoretic Light Scattering
D_H_: Hydrodynamic diameter
ζ_P_: Zeta Potential
GAPDH: Glyceraldehyde 3-phosphate dehydrogenase
ANOVA: Analysis of variance
