# Correction: Vibropolyfection: coupling polymer-mediated gene delivery to mechanical stimulation to enhance transfection of adherent cells

**DOI:** 10.1186/s12951-022-01690-5

**Published:** 2022-11-19

**Authors:** Federica Ponti, Nina Bono, Luca Russo, Paolo Bigini, Diego Mantovani, Gabriele Candiani

**Affiliations:** 1 genT_LΛB, Department of Chemistry, Materials and Chemical Engineering “G. Natta”, Politecnico di Milano, Milan, Italy; 2 Laboratory for Biomaterials and Bioengineering, CRC Tier I, Department of Min-Met-Mat Engineering and CHU de Québec Research Center, Division of Regenerative Medicine, Laval University, Quebec, QC Canada; 3 Department of Molecular Biochemistry and Pharmacology, Istituto di Ricerche Farmacologiche Mario Negri, IRCCS, Milan, Italy

## Correction: Journal of Nanobiotechnology (2022) 20:363https://doi.org/10.1186/s12951-022-01571-x

Following publication of the original article [1], the authors would like to replace the references [64] and [65] with the following references:

- Roffay, Chloé, et al. "Passive coupling of membrane tension and cell volume during active response of cells to osmosis." Proceedings of the National Academy of Sciences 118.47 (2021): e2103228118. https://doi.org/10.1073/pnas.2103228118
- Moe, Alison M., Adriana E. Golding, and William M. Bement. "Cell healing: Calcium, repair and regeneration." Seminars in cell & developmental biology. Vol. 45. Academic Press, 2015. https://doi.org/10.1016/j.semcdb.2015.09.026”
