# Supplementary material for: Vibropolyfection: coupling polymer-mediated gene delivery to mechanical stimulation to enhance transfection of adherent cells
Source: J Nanobiotechnology. 2022 Aug 6;20:363. doi: 10.1186/s12951-022-01571-x (PMC9356458; doi:10.1186/s12951-022-01571-x)

**Supplementary Information**

**Title:**

Vibropolyfection: coupling polymer-mediated gene delivery to mechanical stimulation to enhance transfection of adherent cells

**Authors List:**

Federica Ponti^1, 2, §^

Nina Bono^1, §^

Luca Russo^3^

Paolo Bigini^3^

Diego Mantovani^2^

Gabriele Candiani^1,^ *

**Affiliations:**

^1^ genT_LΛB, Department of Chemistry, Materials and Chemical Engineering “G. Natta”, Politecnico di Milano, Milan, Italy

^2^ Laboratory for Biomaterials and Bioengineering, CRC Tier I, Department of Min-Met-Mat Engineering and CHU de Québec Research Center, Division of Regenerative Medicine, Laval University Québec, QC, Canada

^3^ Department of Molecular Biochemistry and Pharmacology, Istituto di Ricerche Farmacologiche Mario Negri, IRCCS, Milan, Italy

**Corresponding Author:**

* Gabriele Candiani

genT_LΛB, Department of Chemistry, Materials and Chemical Engineering “G. Natta”, Politecnico di Milano, Milan, Italy.

**Tel.** +39 0223993181 **Fax** +390223993180

**E-mail**: gabriele.candiani@polimi.it

§ These authors equally contributed to this work.

**Table of contents**

[Figure S1. Effect of vibrational loading on the viability and morphology of HeLa and MG-63 cells, and bAPCs. 3](#_Toc107594613)

[Table S1. Evaluation of bleb size in vibrational-loaded cells. 4](#_Toc107594614)

[Figure S2. Effect of the cell stimulation on the transfection efficiency and cytotoxicity of *l*PEI/pGL3 and *b*PEI/pGL3 complexes. 5](#_Toc107594615)

[Figure S3. Comparative transfection efficiencies of *l*PEI/pGL3 and *b*PEI/pGL3 complexes delivered before mechanical cell stimulation. 6](#_Toc107594616)

[Figure S4. Effect of vibrational loading on the physicochemical features of polyplexes. 7](#_Toc107594617)

[Figure S5. Evaluation of the effect of vibrational loading on naked pDNA delivery. 8](#_Toc107594618)

[Figure S6. Effect of vibrational loading on cell membrane permeability. 9](#_Toc107594619)

[Figure S7. Effect of the vibrational loading on the cytotoxicity and transfection efficiency of *l*PEI/pGL3 and *b*PEI/pGL3 polyplexes on Jurkat cell suspensions. 10](#_Toc107594620)

[Figure S8. Effect of two endocytosis inhibitors on the viability of polyfected and vibropolyfected L929 cells. 11](#_Toc107594621)

**Figure S1.** Effect of vibrational loading on the viability and morphology of HeLa and MG-63 cells, and bAPCs. (**a**) Viability of HeLa and MG-63 cells, and bAPCs after the application of a 5 min-long vibrational loading at f_in_ = 100 Hz, 500 Hz, and 1,000 Hz. Results are expressed as mean ± SD (n ≥ 3). Unstimulated cells were kept as controls, and their viability (100 %) as baseline. Representative SEM micrographs of (**b**) HeLa, (**c**) MG-63 and (**d**) bAPCs after stimulation under the above conditions. Unstimulated cells (controls) were used as reference standards. Larger background images were taken at ×1,500 (scale bar = 20 µm) and inset micrographs at ×7,000 magnification (scale bar = 5 µm)**.**


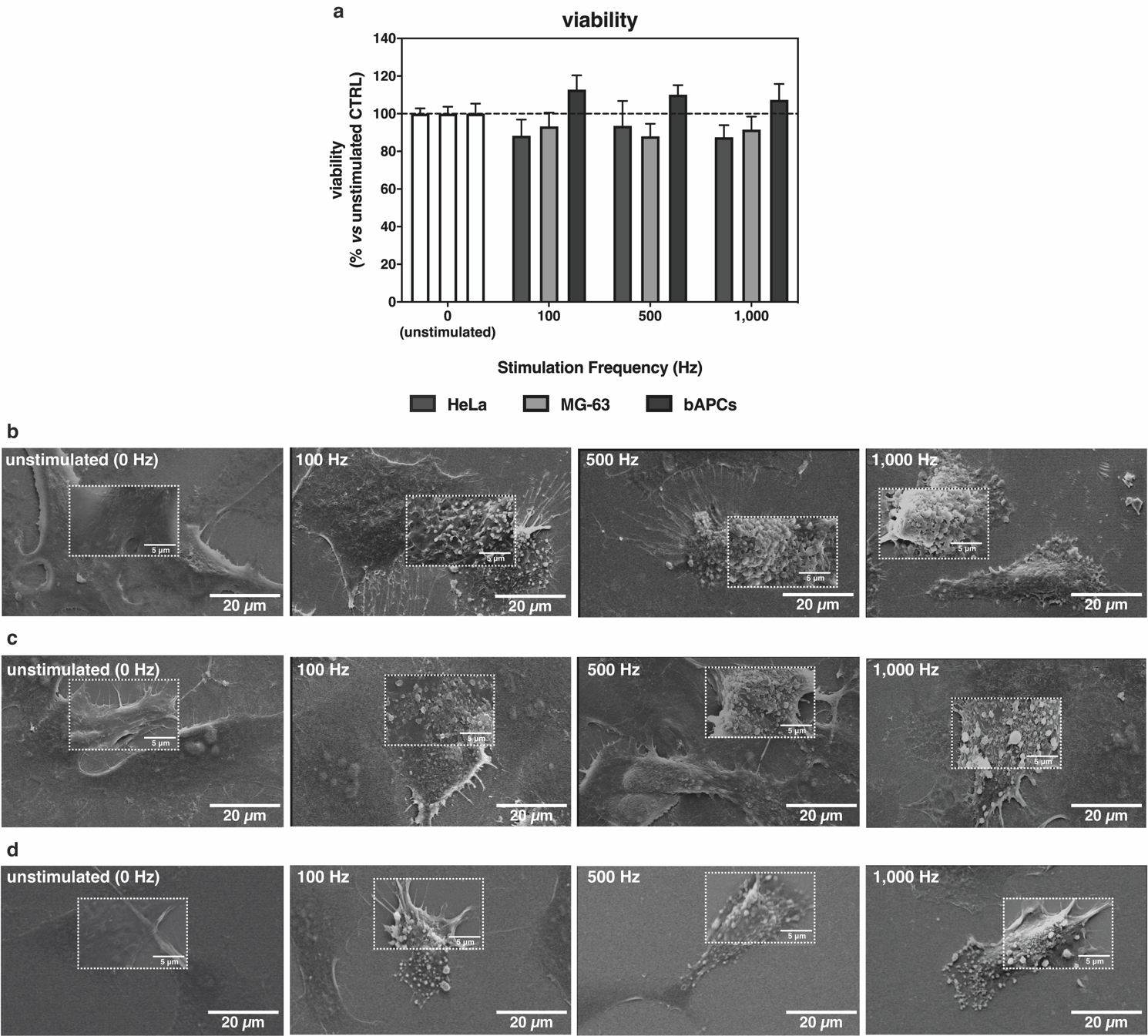


**Table S1.** Evaluation of bleb size in vibrational-loaded cells. Mean bleb diameter (expressed in µm) measured on vibrated cells at f_in_ = 100 Hz, 500 Hz, and 1,000 Hz for 5 min. The mean diameter values were obtained by measuring the average bleb area with Image J analysis on three different images for each condition. Results are expressed as mean ± SD.

|  |  | **cell type** | | | |
| --- | --- | --- | --- | --- | --- |
|  |  | **L929** | **HeLa** | **MG-63** | **bAPCs** |
| **f_in_** | **100 Hz** | 1.14 ± 0.3 | 0.72 ± 0.1 | 0.82 ± 0.1 | 0.81 ± 0.2 |
|  | **500 Hz** | 0.99 ± 0.2 | 0.88 ± 0.2 | 0.71 ± 0.1 | 1.00± 0.3 |
|  | **1,000 Hz** | 1.14 ± 0.2 | 0.92 ± 0.2 | 0.97 ± 0.3 | 0.90 ± 0.2 |

**Figure S2.** Effect of the cell stimulation on the transfection efficiency and cytotoxicity of *l*PEI/pGL3 and *b*PEI/pGL3 complexes. Twenty-four hrs post-seeding, HeLa and MG-63 cells, and bAPCs were challenged with polyplexes, stimulated for 5 min at different frequencies (f_in_ = 100 Hz, 500 Hz, and 1,000 Hz), then cultured for 24 hrs in standard conditions. Cytotoxicity and transfection efficiency were assessed 24 hrs post-polyplex delivery. (**a-d**) Transfection efficiency expressed in terms of (**a, b**) modified firefly luciferase activity normalized to the total protein content (RLU/mg of protein) and (**c, d**) fold increase in transfection efficiency for vibropolyfection conditions over standard transfections (static; polyfection), and (**e, f**) cytotoxicity is the percent cell toxicity (%) as compared to unstimulated and untransfected cells (CTRL). (**a, c, e**) refer to *l*PEI/pGL3 (black bars), while (**b, d, f**) to *b*PEI/pGL3 (gray bars) complexes. Each number within bars in the panels (**c)** and (**d)** refers to the average fold increase value of the dataset. Results are expressed as mean ± SD (n ≥ 3; * p < 0.05 *vs.* unstimulated cells, # p < 0.05 *vs.* 100 Hz).


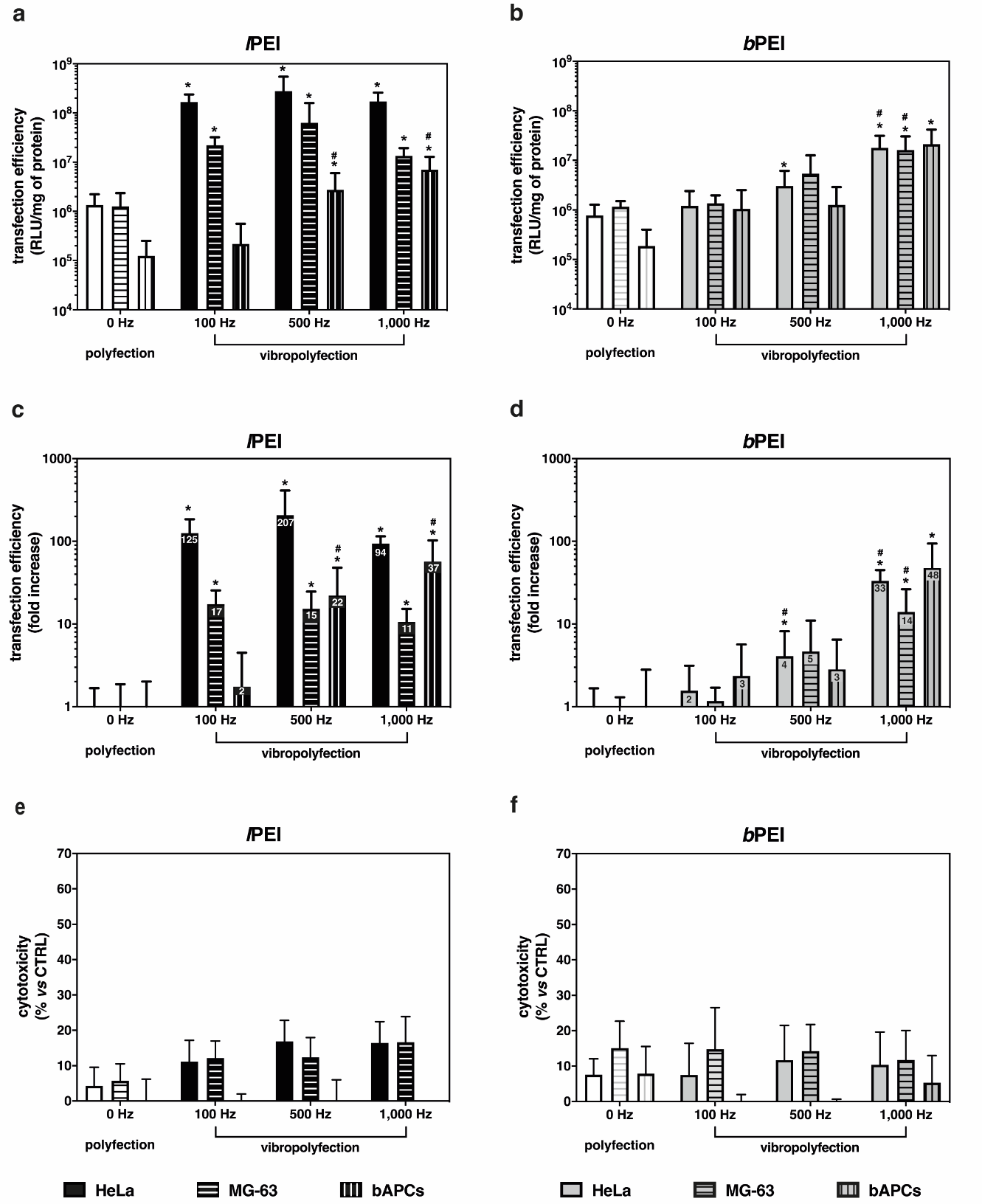


**Figure S3.** Comparative transfection efficiencies of *l*PEI/pGL3 and *b*PEI/pGL3 complexes delivered before mechanical cell stimulation. Twenty-four hrs post-seeding, L929 cells were challenged with polyplexes, stimulated for 5 min at different frequencies (f_in_ = 100 Hz, 500 Hz, and 1,000 Hz), then grown for 24 hrs in standard culture conditions. Cytotoxicity and transfection efficiency were assessed 24 hrs post-polyplex delivery. (**a-d**) Transfection efficiency expressed in terms of (**a, b**) modified firefly luciferase activity normalized to the total protein content (RLU/mg of protein) and (**c, d**) fold increase in transfection efficiency for vibropolyfection conditions over standard transfections (static; polyfection), and (**e, f**) cytotoxicity is the percent cell toxicity (%) as compared to unstimulated and untransfected cells (CTRL). (**a, c, e**) refer to *l*PEI/pGL3 (black bars), while (**b, d, f**) to *b*PEI/pGL3 (gray bars) complexes. Each number within bars in the panels (**c)** and (**d)** refers to the average fold increase value of the dataset. Results are expressed as mean ± SD (n ≥ 3; * p < 0.05).


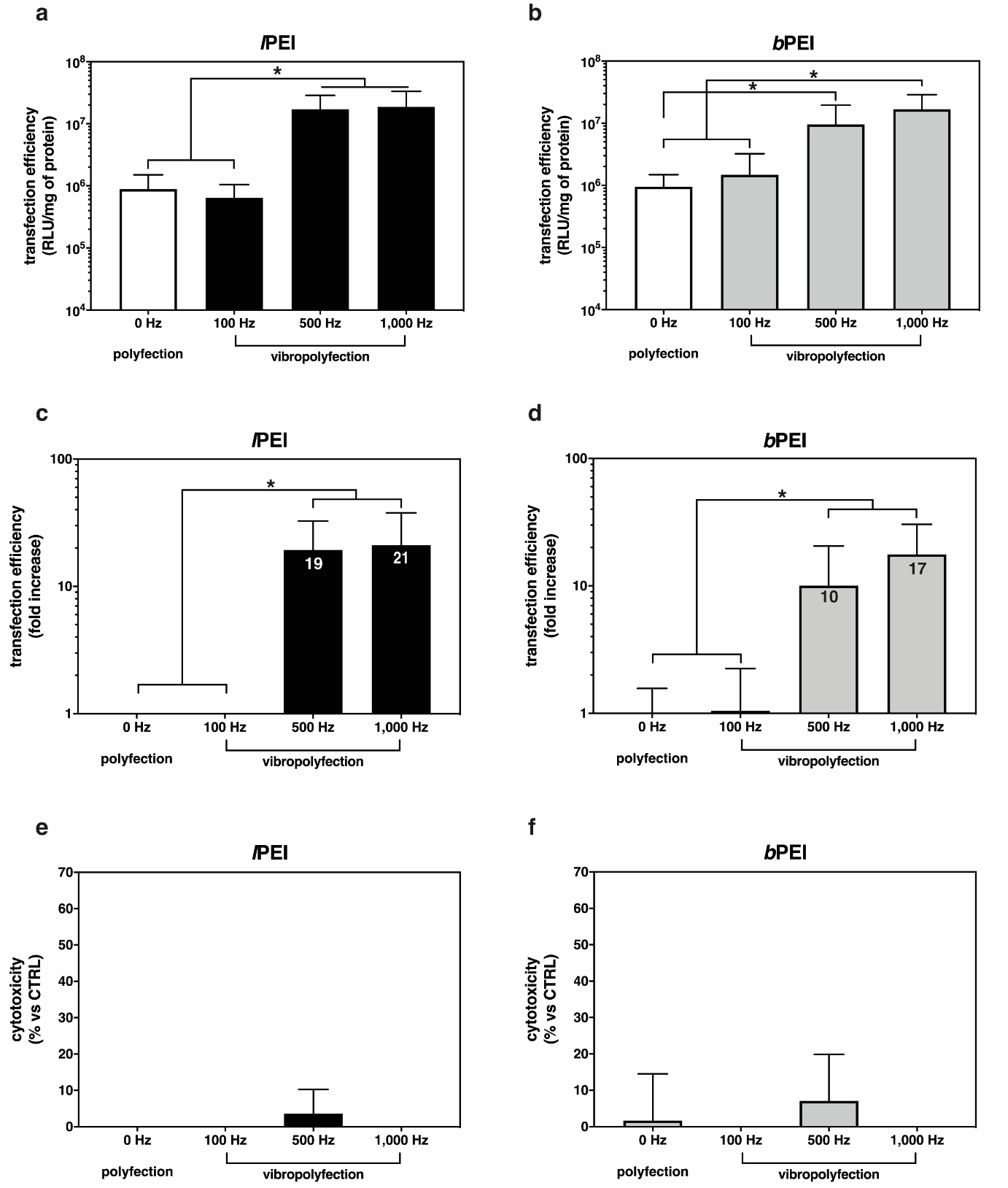


**Figure S4.** Effect of vibrational loading on the physicochemical features of polyplexes. Mean hydrodynamic diameter (D_H_; black dots) and zeta-potential (ζ_P_; red squares) of (**a**) *l*PEI/pDNA and (**b**) *b*PEI/pDNA complexes in 10 mM HEPES after vibrational loading for 5 min at f**_in_** = 100 Hz, 500 Hz, and 1,000 Hz. Results are expressed as mean ± SD (n = 3).


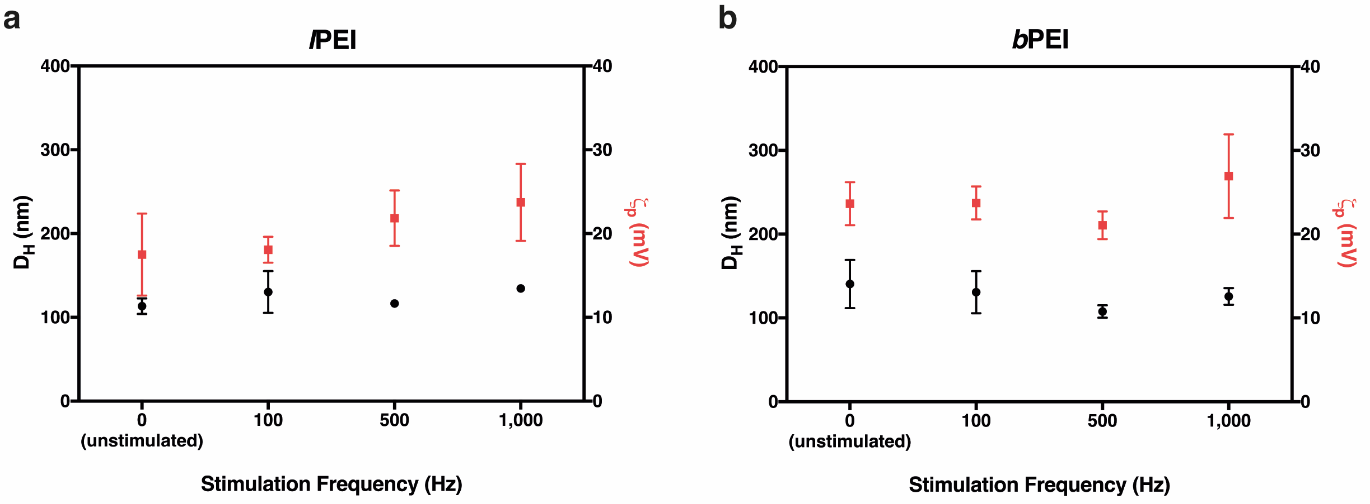


**Figure S5**. Effect of vibrational loading on naked pDNA delivery. Briefly, 24 hrs post-seeding, L929 cells were challenged with either pristine pGL3 (0.16 µg/cm^2^) or *l*PEI/pGL3 or *b*PEI/pGL3 complexes. Cells were either kept under static conditions (unstimulated, 0 Hz) or stimulated at f_in_ = 1,000 Hz for 5 min, then grown for 24 hrs in standard culture conditions. Transfection efficiency was assessed after 24 hrs post-pGL3 or polyplex delivery. Transfection efficiency is expressed as modified firefly luciferase activity normalized to the total protein content (RLU/mg of protein). Results are expressed as mean ± SD (n = 3, * p < 0.05).


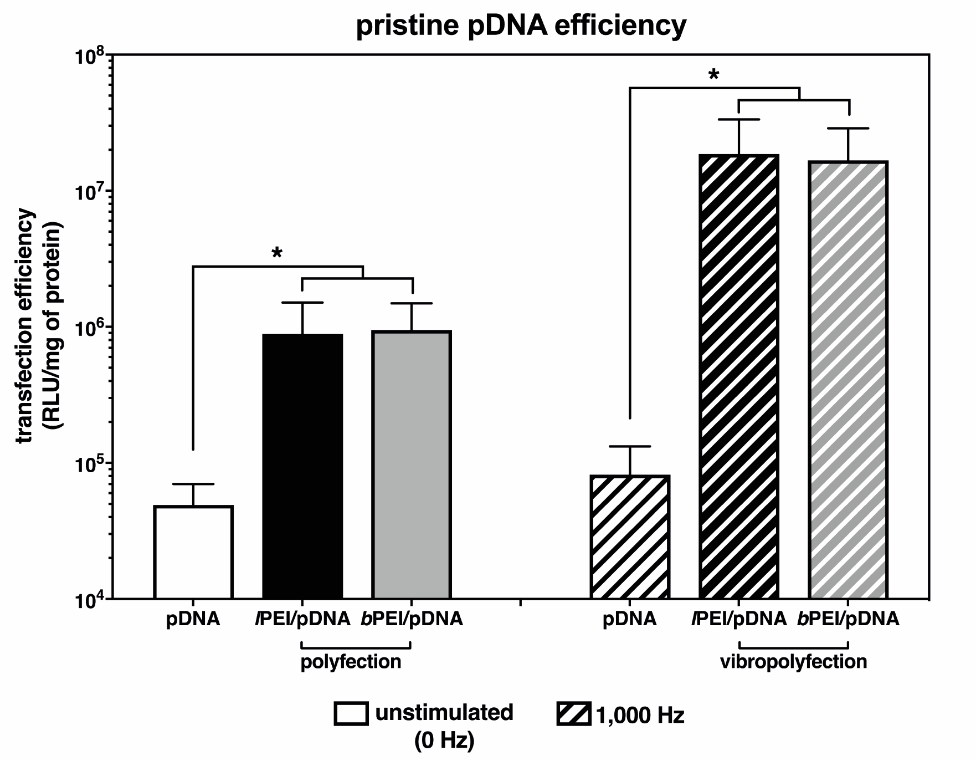


**Figure S6.** Effect of vibrational loading on cell membrane permeability. L929 cells were incubated with Trypan Blue dye before being exposed to vibrational loading for 5 min at f_in_ = 1,000 Hz. Unstimulated cells incubated with the Trypan Blue dye for 5 min were taken as negative controls of permeabilization. Positive control of permeabilization were cells treated for 5 min with 0.01 % Triton X-100, next incubated with the Trypan Blue dye. Results are expressed as Trypan Blue-positive cells (%), represented as mean ± SD (n = 3; * p < 0.05).


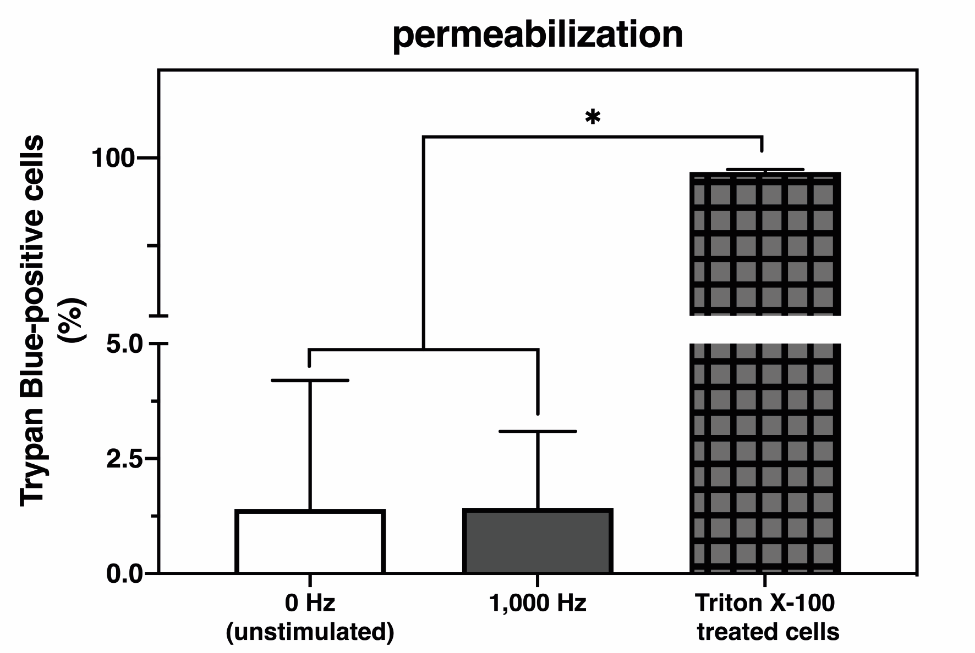


**Figure S7.** Effect of the vibrational loading on the cytotoxicity and transfection efficiency of *l*PEI/pGL3 and *b*PEI/pGL3 polyplexes on Jurkat cell suspensions. Briefly, Jurkat cells were challenged with *l*PEI/pGL3 and *b*PEI/pGL3 polyplexes according to polyfection and vibropolyfection protocols (post-delivery set-up, f_in_ = 1,000 Hz) and cultured in standard conditions for 24 hrs. (**a**) Cytotoxicity and (**b**) transfection efficiency were assessed 24 hrs post-addition of polyplexes. Cytotoxicity is expressed as percent cell toxicity (%) as compared to unstimulated and untransfected cells (CTRL), while transfection efficiency is expressed as modified firefly luciferase activity normalized to the total protein content (RLU/mg of protein). Results are expressed as mean ± SD (n = 3).


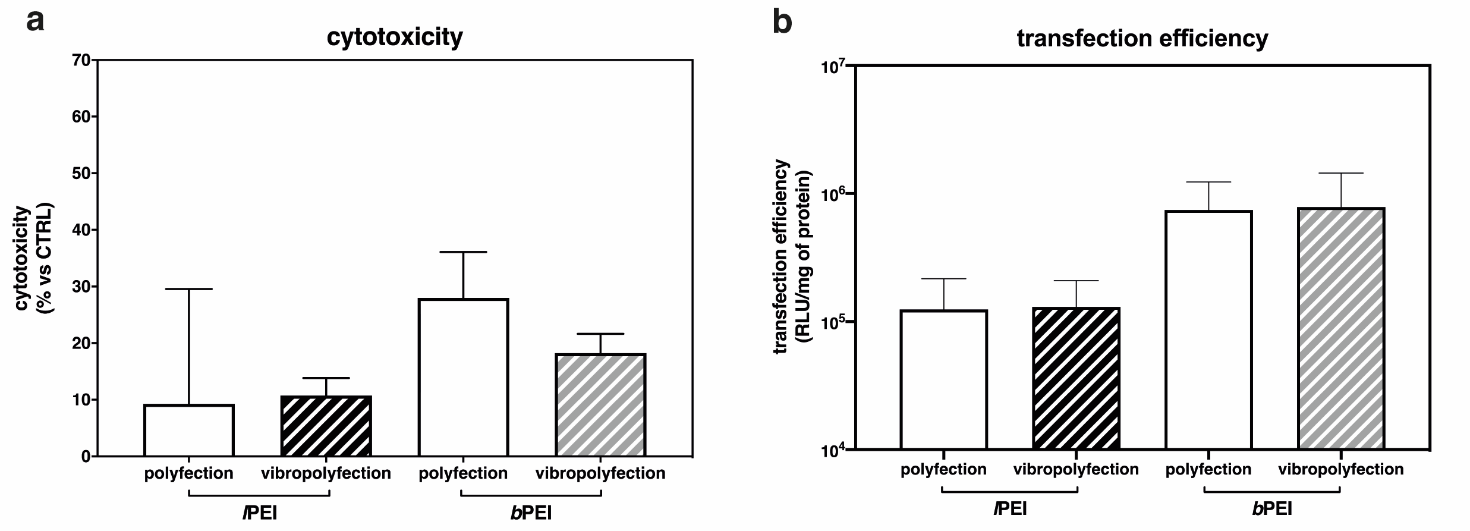


**Figure S8.** Effect of two endocytosis inhibitors on the viability of polyfected and vibropolyfected L929 cells. Briefly, 24 hrs post-seeding in cell culture plates, cells were incubated for 30 min with clathrin and caveolae inhibitors, named chlorpromazine (10 µg/mL) and filipin (5 µg/mL). Next, the medium was replaced, and the cells were either cultured for 24 hrs in standard conditions or subjected to a 5 min-long vibrational loading at f_in_ = 1,000 Hz before being cultured. The cytotoxic effect of inhibitors was assessed 24 hrs post-treatment. Cytotoxicity is expressed as non-viable cells (%) to matched untreated controls. Results are expressed as mean ± SD (n = 3).


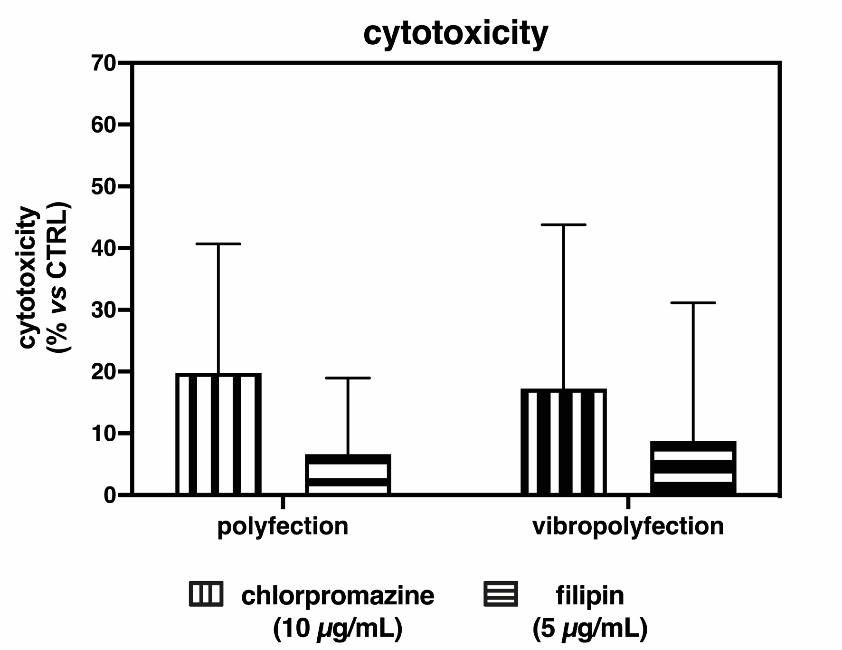

Supplement: Supplementary file 1 — Additional file 1: Figure S1. Effect of vibrational loading on the viability and morphology of HeLa and MG-63 cells, and bAPCs. Table S1. Evaluation of bleb size in vibrational-loaded cells. Figure S2. Effect of the cell stimulation on the transfection efficiency and cytotoxicity of lPEI/pGL3 and bPEI/pGL3 complexes. Figure S3. Comparative transfection efficiencies of lPEI/pGL3 and bPEI/pGL3 complexes delivered before mechanical cell stimulation. Figure S4. Effect of vibrational loading on the physicochemical features of polyplexes. Figure S5. Evaluation of the effect of vibrational loading on naked pDNA delivery. Figure S6. Effect of vibrational loading on cell membrane permeability. Figure S7. Effect of the vibrational loading on the cytotoxicity and transfection efficiency of lPEI/pGL3 and bPEI/pGL3 polyplexes on Jurkat cell suspensions. Figure S8. Effect of two endocytosis inhibitors on the viability of polyfected and vibropolyfected L929 cells. [file 12951_2022_1571_MOESM1_ESM.docx]
